# Programmed Cell Death in Chronic Rhinosinusitis

**DOI:** 10.1002/clt2.70175

**Published:** 2026-06-02

**Authors:** Xingchen Liu, Junying Hu, Weigang Gan, Qi Wu, Bing Zhong, Feng Liu

**Affiliations:** ^1^ Department of Otolaryngology Head and Neck Surgery West China Hospital of Sichuan University Chengdu Sichuan Province China

**Keywords:** apoptosis, chronic rhinosinusitis, necroptosis, programmed cell death, pyroptosis

## Abstract

Chronic rhinosinusitis (CRS) is a prevalent upper respiratory condition characterized by a multifaceted etiology involving various cellular and molecular processes. In recent years, researchers have increasingly recognized the significance of different forms of programmed cell death (PCD), such as apoptosis and pyroptosis, in the pathological mechanisms underlying CRS. Studies suggest that these PCD pathways not only influence the disease's progression but may also serve as novel therapeutic targets. Thus, gaining a thorough understanding of how PCD contributes to CRS is crucial for elucidating its pathogenesis and developing new treatment strategies. This article aims to investigate the mechanisms of PCD in CRS, examining its effects on disease progression and potential implications for treatment. We will begin by discussing the background of CRS and its underlying processes, followed by an exploration of the roles played by various types of PCD in CRS, and conclude with a discussion on future research directions and their practical applications.

**Abbreviations**
AAT: alpha‐1 Antitrypsin
AATD: alpha‐1 Antitrypsin Deficiency
AIM2: absent in Melanoma 2
AQP4: aquaporin 4
AR: allergic Rhinitis
BAX/BAK: BCL‐2‐Associated X Protein/BCL‐2 Antagonist Killer 1
BCL‐2: B‐cell Lymphoma 2
cIAP1/2: cellular inhibitor of apoptosis protein 1/2
CRS: chronic rhinosinusitis
CRSsNP: chronic rhinosinusitis without nasal polyps
CRSwNP: chronic rhinosinusitis with nasal polyps
DAMPs: damage‐associated molecular patterns
DDX33/DDX58: DEAD‐Box Helicase 33/58
DNase I: deoxyribonuclease I
ECP: eosinophil cationic protein
ECRS: eosinophilic chronic rhinosinusitis
ERK: extracellular signal‐regulated kinase
ETosis: extracellular trap cell death
Fas/Fas‐L: fas ligand
GSDMD: gasdermin D
HIF‐1α: hypoxia‐inducible factor 1‐alpha
HNECs: human nasal epithelial cells
IAP: inhibitor of apoptosis protein
IFN‐γ: interferon‐gamma
IL‐1β/IL‐18: interleukin‐1β/interleukin‐18
JNK: c‐jun N‐terminal kinase
LCN2: lipocalin‐2
MAPK: mitogen‐activated protein kinase
MBP: major basic protein
MLKL: mixed lineage kinase domain‐like
NLRP3: NOD‐like receptor family pyrin domain containing 3
PD‐1/PD‐L1: programmed death‐1/programmed death‐ligand 1
RAGE: receptor for advanced glycation end‐products
RIPK1/RIPK3: receptor‐interacting protein kinase 1/3
ROS: reactive oxygen species
RUNX1: runt‐related transcription factor 1
STAT5: signal transducer and activator of transcription 5
TLR4: toll‐like receptor 4
TNF‐α: tumor necrosis factor‐alpha
uPA/uPAR: urokinase‐type plasminogen activator/uPA receptor
VEGF: vascular endothelial growth factor
XIAP: X‐linked inhibitor of apoptosis protein

## 1 Introduction

Chronic rhinosinusitis (CRS) is a prevalent inflammatory condition that affects the upper respiratory tract worldwide, characterized by persistent inflammation of the nasal cavity and sinus mucosa [1]. Typical pathological features of CRS include thickened mucosal lining, gland hyperplasia, and changes in epithelial cells, along with the infiltration of inflammatory cells [2]. CRS can be categorized into two types based on the inflammatory response: eosinophilic chronic rhinosinusitis (ECRS) and non‐eosinophilic chronic rhinosinusitis (non‐ECRS) [3]. ECRS is associated with severe symptoms and higher recurrence risk after treatment, mainly due to eosinophils releasing cytotoxic proteins that damage tissue [4].

Furthermore, the abnormal accumulation and dysfunction of immune cells in the mucosa of CRS patients may be closely associated with the dysregulation of programmed cell death (PCD) [5]. Recent studies indicate PCD significantly influences CRS onset and progression. PCD is a regulated cell death process essential for maintaining cellular balance and tissue function by removing unnecessary cells and allowing regeneration [6, 7]. Unlike necrosis, PCD is orderly and energy‐dependent, involving complex signaling and gene regulation. Important forms of PCD, such as apoptosis, necroptosis, and pyroptosis [8], play a role in regulating inflammation and are receiving increased attention in CRS research.

Apoptosis, described as “cell suicide” is a vital PCD process that maintains tissue balance and removes damaged or dysfunctional cells [9]. It involves controlled biochemical changes, such as membrane folding and nucleus fragmentation, leading to orderly cell death. This process can be triggered by external signals like cytokine absence or internal factors like DNA damage and oxidative stress [10]. Caspases regulate apoptosis by activating molecules that cause cell death [11]. In CRS, regulating the clearance of inflammatory immune cells is crucial to control inflammation. Resistance to apoptosis, often due to BCL‐2 overexpression, leads to cell accumulation [9], while normal cells may develop resistance to protect against inflammation.

Necroptosis is a type of PCD that occurs due to significant damage or infection. Unlike apoptosis, it involves necroptotic bodies and leads to cell membrane rupture, releasing cellular contents that trigger immune responses [12]. It is regulated by RIP kinases 1 and 3, with the RIPK pathway involved in infections, ischemia, and tumors [13]. This complex regulation interacts with NF‐κB and MAPK pathways, crucial for inflammation [12]. In CRS, necroptosis may exacerbate inflammation, leading to worsened symptoms and reduced treatment efficacy [14].

Pyroptosis is PCD associated with inflammation and various diseases, characterized by inflammasomes activation and caspase‐1, which results in rapid cell death and the release of inflammatory factors [9]. It is prevalent in bacterial infections and autoimmune disorders, compromising cell membrane integrity and triggering inflammation [15]. Recent studies highlight the role of pyroptosis in disease progression, particularly in tumors, as it contributes to immune evasion and treatment resistance [16]. In CRS, pyroptosis can worsen symptoms and lead to additional tissue damage by increasing local inflammation, underscoring its critical role in CRS pathogenesis [17].

**FIGURE 1:**
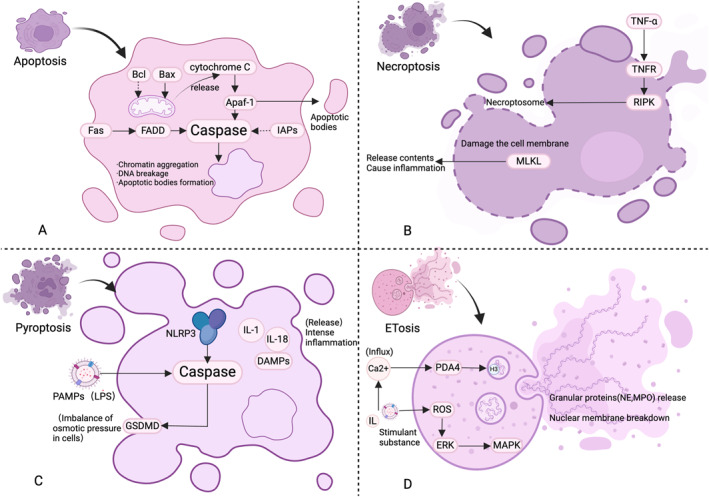
Molecular network regulating apoptosis, necroptosis, and pyroptosis in multiple cell types. (A) Regulatory axes of intrinsic and extrinsic apoptosis pathways and their related factors in cells. (B) Mechanistic diagram of necroptosis in cells. (C) Mechanism of pyroptosis in cells (NLRP3 inflammasome pathway). (D) ETosis mechanism pathway in cells.

ETosis is a specialized form of cell death triggered by certain immune cells, marked by the release of extracellular traps (ETs) that serve to capture and eliminate microorganisms. This process has recently been recognized as a type of PCD [18]. Research shows eosinophils and macrophages can also release ETs [19]. This process involves the decondensation of chromatin to form DNA and antimicrobial protein networks that neutralize pathogens. In conditions like CRS, ETosis may significantly influence the progression of the disease by affecting local immune responses [20] (Figure 1).

A deeper understanding of PCD in chronic respiratory conditions reveals disease mechanisms and the “inflammation‐injury‐remodeling” cycle. The interaction network of PCD subtypes in CRS involves mucus hypersecretion. Th2 responses, and tissue remodeling, requiring further examination of timing and location. The research summarizes the relationship between PCD components and CRS, aiming to develop targeted therapies like Bcl‐2 and necroptosis inhibitors for clinical applications. Table 1 summarizes key factors and pathways of programmed cell death in CRS.

**TABLE 1:** Key stimulating factors and signaling pathways of PCD in CRS.

PCD type	Stimulating factors	Primary cell types	Key signaling pathways/mechanisms (sentence‐level citations)
Apoptosis	Host‐derived stimuli/regulators: Cytokine milieu and death‐receptor signaling Oxidative stress/smoke exposure ER stress/DNA damage Anti‐apoptotic programs	HNECs; HNEpCs; eosinophils; fibroblasts; macrophages; T cells	Fas/Fas‐L and TRAIL–TRAILR2 signaling stimulate epithelial apoptosis [21]. IFN‐γ inhibits apoptosis and regulates TRAIL receptor expression [22]. IFN‐γ regulates epithelial cell fate through p62‐dependent autophagy signaling [23]. Oxidative stress activates RAGE–p38 MAPK signaling, promoting ROS production and tissue injury [24]. PTEN mediates H_2_O_2_‐induced apoptosis, while caspase‐3/9 execute mitochondrial apoptosis [25, 26]. Enhanced Bcl‐2 family activity inhibits apoptosis and promotes persistent inflammation [27].L
	Pathogens: *S*. *aureus* Respiratory viruses	HNECs; immune cells	SpA can engage TNFR1‐associated pro‐apoptotic signaling in airway epithelial contexts [28]. HRV 3C protease can disrupt the RIPK1–TRIF/FADD/SQSTM1 complex, thereby attenuating apoptosis‐related signaling [29]. Suppressing anti‐apoptotic proteins (e.g., Mcl‐1/Bcl‐xL)in respiratory infections can lead to mitochondrial apoptosis and pyroptosis [30].
Necroptosis	Host‐derived stimuli/regulators: TNF‐α + IFN‐γ co‐stimulation ER stress–CHOP signaling IL‐1β–enriched inflammatory milieu	HNECs; macrophages; other immune cells	TNF‐α and IFN‐γ co‐stimulation activates the RIPK1–RIPK3–MLKL pathway, inducing necroptosis [31]. ER stress activates CHOP signaling, regulating macrophage necroptotic fate [32]. Necroptosis releases DAMPs, promoting inflammatory amplification and tissue remodeling [31].
	Pathogens/colonization: *Staphylococcus* aureus–linked inflammatory triggers Microbiome modulation	HNECs; epithelial–immune interface	Pathogen cues may enhance inflammation by promoting necroptosis when caspases are inhibited. [33]. *Staphylococcus* epidermidis reduces IL‐33 and GATA3 in HNECs, modulating necroptosis‐linked alarmin output [34, 35]..
Pyroptosis	Host‐derived cytokines/intrinsic regulators/oxidative stress: IL‐8, IL‐17A, IL‐1β HMGB1 ROS/hypoxia signaling	HNECs; nasal epithelial cells (NEpCs); Treg; macrophages	IL‐8 and IL‐17A induce HNEC pyroptosis via the ERK–NLRP3/caspase‐1 axis, leading to GSDMD pore formation and IL‐1β/IL‐18 release. [17, 36]. HMGB1 stimulates HNECs to produce IL‐6 and IL‐8, potentially amplifying local inflammatory loops [37]. Hypoxia/oxidative stress can stabilize NLRP3 (via HIF‐1α) and promote inflammasome‐driven epithelial injury [38]. KLF4 elevation increases NLRP3 expression and worsens pyroptosis symptoms in nasal epithelium models [39]. ROS‐driven mitochondrial dysfunction can engage the caspase‐9/3 cascade, linking pyroptosis‐associated stress to apoptotic execution [26].
	Pathogens: *S*. *aureus* HRV/virus	HNECs; macrophages	AIM2/CASP5/NLRP6 has been implicated in pathogen‐associated epithelial pyroptosis [40]. HRV infection can trigger IL‐1β secretion and epithelial pyroptosis via the DDX33/DDX58–NLRP3–caspase‐1–GSDMD pathway [41]. Viral inhibition of Bcl‐2 proteins can induce GSDME‐dependent pyroptosis in airway epithelial cells, with parallels in CRS [30, 42].
ETosis	Host‐derived cytokines/drivers: IL‐5, IL‐1β, IL‐8 ROS–NADPH oxidase axis	Neutrophils; eosinophils; macrophages	NET release involves chromatin decondensation and formation of DNA–antimicrobial protein networks [20, 43]. NETosis is largely ROS dependent and requires NADPH oxidase–derived ROS signaling [20, 44]. NET abundance is linked to neutrophil infiltration, and LL‐37 promotes NET formation [45].
	Pathogens/danger signals: *Staphylococcus aureus* colonization HRV‐associated triggers cfDNA–TLR9 signaling	HNECs; polyp tissue; eosinophils	Eosinophil ETosis captures pathogens and is associated with IL‐5–linked Th2 inflammation and Charcot–Leyden crystals [46, 47]. Cell‐free DNA can activate TLR9 signaling to trigger eosinophil extracellular trap formation [48]. LL‐37 induces HNEC and macrophage death via caspase‐1/‐8 activation and is linked to ET formation [45, 49].

## 2 Apoptosis

### 2.1 Apoptosis in Epithelial Cell

Apoptosis causes loss of human nasal epithelial cells (hNECs), impairing barrier function and increasing infection risk. Ineffective removal of apoptotic cells increases inflammation, damaging sinonasal mucosa [50]. When cells evade apoptosis, they can survive longer and proliferate excessively, whereas the death of inflammatory cells may help reduce inflammation. Research indicates CRS phenotypes can be identified by apoptosis levels in mucosal tissues, as apoptotic bodies contain information from deceased cells [51]. Analyzing microvesicles may help identify CRS phenotypes. Study showed microvesicles (MP) from activated or apoptotic cells show no significant difference between Chronic Rhinosinusitis with Nasal Polyps (CRSwNP) patients and controls, but have less epithelial damage than CRSsNP and aspirin‐exacerbated respiratory disease patients [52].

**FIGURE 2:**
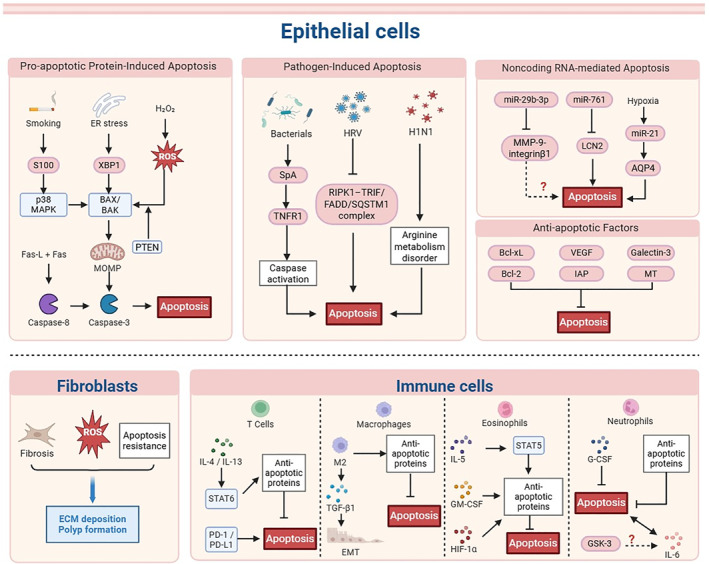
Molecular mechanisms regulating apoptosis in CRS. The diagram summarizes apoptosis regulation across epithelial cells, fibroblasts, and immune cells in CRS. In epithelial cells, apoptosis is driven by (1) pro‐apoptotic protein–induced mitochondrial pathways involving ROS, XBP1, BAX/BAK, and caspase‐3 activation; (2) pathogen‐induced signaling triggered by bacterial and viral factors such as *Staphylococcus aureus* protein A, HRV, and H1N1; and (3) noncoding RNA–mediated regulation involving miR‐29b‐3p, miR‐761, and miR‐21. Anti‐apoptotic factors including Bcl‐2, Bcl‐xL, IAPs, VEGF, Galectin‐3, and metallothionein suppress apoptosis and promote epithelial survival. In fibroblasts, ROS accumulation and apoptosis resistance contribute to ECM deposition and polyp formation. In immune cells, apoptosis is regulated by cytokine‐mediated signaling pathways including STAT6, STAT5, PD‐1/PD‐L1, and G‐CSF‐related pathways, which influence the survival of T cells, macrophages, eosinophils, and neutrophils and contribute to persistent inflammation in CRS.

Currently, there is ongoing debate regarding apoptosis in CRS epithelial tissue. Cheng reported reduced goblet cell apoptosis in a CRS mouse model [53], and decreased levels of apoptotic proteins have also been observed in nasal polyps, suggesting reduced apoptosis in this context [54]. In contrast, Hu noted high apoptosis and low growth in CRSsNP hNECs [55], and Morawska reported increased apoptosis in CRSwNP epithelial cells [56] (Figure 2).

These discrepancies likely arise from non‐equivalent comparisons. Studies differ in population/geographic setting (East Asian vs. European cohorts) [54], sampling site and specimen type (polyp tissue、turbinate、non‐polyp mucosa、maxillary sinus mucosa or cultured primary epithelium), and apoptosis readouts (caspase expression, protein levels, Annexin‐V/PI events, or TUNEL positivity). Therefore, “increased” or “reduced” apoptosis should be interpreted only after specifying CRS phenotype, anatomical sampling, and the assay platform.

#### 2.1.1 Pro‐Apoptotic Factors

**Pro‐Apoptotic Protein**: Pro‐apoptotic proteins induce inflammation and alter tissue structure in CRS by triggering mitochondrial apoptosis. Apoptotic proteins such as XBP1 are elevated in CRSsNP HNECs and induce apoptosis during Endoplasmic reticulum (ER) stress [57]. Levels of uPA and uPAR, which promote apoptosis and cell proliferation, are also increased in CRSsNP [55]. Signal transducer and activator of transcription STAT5a and STAT5b are two closely related kinases involved in regulating various cell functions, including proliferation and apoptosis [58], increased activation in CRSwNP [59]. Moreover, H2O2 elevates reactive oxygen species (ROS) levels, leading to epithelial apoptosis and oxidative stress, an effect that is exacerbated by PTEN [25]. Meanwhile, RAGE activation by S100 proteins in smoking‐related CRS generates mitochondrial ROS via p38 MAPK, triggering BAX/BAK‐dependent apoptosis and inhibiting antioxidant enzymes like SOD, worsening epithelial injury [24]. Emerging proteins like S100A11 are crucial in CRS pathogenesis and may serve as new therapeutic targets by balancing inflammation and apoptosis [60].

P53 is a key tumor suppressor that regulates cell cycle, DNA repair, and apoptosis for genomic stability [61]. P53 upregulates caspase‐3, promoting apoptosis and preventing uncontrolled growth [62]. P53 is extensively studied in NPs, often found in eCRSwNP mucosa [63], and its expression is lower in CRSwNP patients compared to controls [64]. EosCRS has higher p53 and PCNA levels, while the neutrophilic type has lower p53 and p21 levels [65]. DFF45, a caspase‐3 substrate, indicates immune cell‐mediated apoptosis resistance, with higher levels in eosinophilic than in neutrophilic and lymphocytic types [66].

Fas and Fas‐L are cell surface pairs that mediate programmed cell death, influencing immune regulation, tissue homeostasis, and tumor immune evasion [67]. The interaction between Fas and Fas‐L activates caspase signaling, which leads to DNA fragmentation and the dissociation of DFF45 from DFF40 [68]. This apoptosis may contribute to immune privilege in, where Fas‐L positive cells grow subepithelially. Fas‐L gene transcript levels are similar in polyps and nasal mucosa, but protein levels are higher in polyps [69]. Basinski showed that Fas‐Fas ligand and TRAIL‐TRAIL receptor 2 interactions enhance apoptosis in epithelial cells [21]. IFN‐γ inhibits poptosis [22]. CRS hNECs show higher HLA‐DR, IP‐10, MCP, and TRAIL levels. IFN‐γ triggers HLA‐DR, TRAIL, and TNF receptor 2 but decreases TRAIL receptor 4 [43]. TRAIL and TRAIL receptor 2 interaction enhances apoptosis, and IFN‐γ activates autophagy [23], insufficiently causing p62‐dependent apoptosis in CRSwNP.

**Noncoding RNA**: miRNA regulates the cell cycle and apoptosis. In the nasal epithelium of CRSwNP, levels of miR‐203a‐3p, miR‐205‐5p, miR‐221‐3p, miR‐222‐3p, miR‐378a‐3p, miR‐449a, and miR‐449b‐5p rise, while levels of miR‐17‐5p and miR‐145‐5p decrease [56, 70]. Liu noted that increased miR‐29b‐3p enhances α‐tubulin deacetylation by lowering MMP‐9‐integrinβ1 complexes, influencing apoptosis [71]. Low levels of miR‐761 activate LCN2, leading to the inhibition of CRS cell growth and the promotion of apoptosis [72]. CRS hNECs affect apoptosis via the HIF‐1α‐miR‐21‐AQP4 axis [73].

**Bacteria and Viruses**: Bacterial and viral infections can cause apoptosis in HNECs, which damages the sinonasal mucosa [74]. *Staphylococcus aureus* (*S*. *aureus*), a common pathogen in CRS, causes apoptosis and a strong pro‐inflammatory response in nasal tissue, triggered by bacterial components and biofilm matrix [75]. *Staphylococcus aureus* protein A (SpA) on membranes can induce apoptosis in HNECs [76], helping bacteria evade the immune system by preventing inflammation [77]. Activating TNFR1 is crucial for pro‐inflammatory and pro‐apoptotic signals. SpA signals through TNFR1 in airway epithelial and immune cells, also activating type I interferon signaling [28].

HRV disrupt the RIPK1‐TRIF/FADD/SQSTM1 immune complex via 3C protease, impairing epithelial cell apoptosis [29]. Additionally, PD‐1/PD‐L1 is crucial for immune evasion during nasal mucosal invasion. They release immunosuppressive cytokines [78], increasing PD‐L1 and PD‐L2 levels in allergic mucosa [79], exacerbating CRS symptoms. H1N1 strains also interfere with arginine metabolism, leading to apoptosis in nasal epithelial progenitor cells and inflammation [80].

#### 2.1.2 Anti‐Apoptotic Factors

**Anti‐Apoptotic Proteins**: Anti‐apoptotic proteins contribute to the chronic nature of CRS by inhibiting the apoptosis of inflammatory cells. Anti‐apoptotic proteins can make CRS chronic by inhibiting inflammatory cell apoptosis. The anti‐apoptotic proteins are crucial for the survival of NP epithelial cells. The overexpression of Bcl‐xL in NP epithelial cells may inhibit cell death, leading to abnormal growth and polyp formation [81]. In CRSwNP patients, reduced apoptosis of epithelial cells is associated with increased Bcl‐2 levels, which correlates with disease recurrence [27]. RUNX1 promotes apoptosis by inhibiting Bcl‐2 and enhancing Bax and Caspase‐3 [42].

Currently, the inhibitor of apoptosis protein (IAP) family has been extensively studied in CRS. These proteins are crucial for PCD and modulating the inflammatory process, which may promote the formation of CRSwNP [82, 83]. In CRSwNP, apoptosis regulation involves various pathways and the IAP family, including cIAP1, cIAP2, XIAP, Livin, and Survivin, which influence apoptosis and inflammation. However, Qiu reported elevated Survivin levels in [84], indicating its role in their development, and Yun noted increased Livin levels in CRSwNP patients [85]. Smac (DIABLO) is an IAP‐binding protein that activates caspases and triggers apoptosis [86]. Studies have shown that IAP can directly bind to caspase‐3 and caspase‐9, regulating apoptosis [82]. Caspase‐3 is present in normal nasal mucosa and polyps, with reduced expression in NP tissues indicating decreased apoptosis [54]. Küpper noted lower p53 and caspases 3 and 9 levels in CRSwNP patients compared to controls [64]. IAPs regulate apoptosis through binding to caspases. In contrast, Livin has a negative correlation with caspase‐3 and Smac, which may inhibit apoptosis and influence HNEC growth and inflammation in CRSwNP. Simultaneously, the research indicated that IL‐4, IL‐17A, and IL‐1β upregulate Livin mRNA in CRS [22].

**Others**: Galectin‐3 prevents apoptosis in the mitochondrial pathway, with significant expression in surface and glandular epithelium [87], while low in epithelial cells. H2BK promotes cell death in but is less expressed in CRSwNP tissues than normal [88]. Metallothionein (MT) protects NP cells from immune‐mediated death [89], and is expressed at higher levels in eosinophilic polyps than in lymphocytes [90]. NP1 is found in CRS epithelial cells, with VEGF promoting HNEC growth and preventing cell death [91].

### 2.2 Apoptosis in Fibroblasts

In CRSwNP, there is notable infiltration of fibroblasts and deposition of ECM [92]. NPDF exhibit active growth and higher ROS levels, with increased apoptosis [93]. Meanwhile, fibroblasts resist apoptosis because of lower BIM expression, resulting in reduced apoptosis, exacerbated fibrosis, and a thicker polyp stroma [94].

### 2.3 Apoptosis in T Cells

Research indicates that in CRS, activated T cells interact with epithelial cells, leading to epithelial activation, pro‐inflammatory functions, apoptosis, and reduced inflammation [21]. PD‐1 inhibits T cell activity and regulates immune responses [95], while PD‐L1's binding to PD‐1 reduces the proliferation of PD‐1 positive cells, inhibits cytokine secretion, and induces apoptosis [96]. Research shows IL‐4/IL‐13 prevent T cell death via STAT6 through the STAT6 signaling pathway, which supports the survival of Th2 cells and contributes to inflammation [97].

### 2.4 Apoptosis in Myeloid Immune Cells

In CRS, macrophages express and secrete TGF‐β1, promoting epithelial remodeling [98], and potentially influencing the levels of apoptosis‐preventing proteins [99]. M2 macrophages in CRS regulate lipid metabolism, which impacts proteins such as Bcl‐xL. This regulation supports epithelial cell survival and promotes polyp growth [100].

### 2.5 Apoptosis in Eosinophils

Studies show eosinophil apoptosis in CRS varies by clinical phenotype. Aspirin allergic rhinosinusitis (AERD) patients have fewer dead cells in than AT patients, and longer AERD duration correlates with lower apoptosis levels [101]. CRS patients show elevated HIF‐1α and survival factors in eosinophils, inhibiting apoptosis and leading to chronic inflammation [102]. High levels of anti‐apoptotic proteins like Bcl‐2 sustain inflammatory cell infiltration and pro‐inflammatory factor release [103]. IL‐5 activates the STAT5 pathway, increasing Mcl‐1 and Bcl‐2, inhibiting eosinophil apoptosis and causing tissue damage [104]. Bcl‐xL inhibits apoptosis through the GM‐CSF/IL‐5 signaling pathway, further contributing to eosinophil survival [105]. Siglec‐8, found on mast cells and eosinophils, can initiate lysosomal apoptosis [106]. These mechanisms are most relevant to eosinophilic endotypes, where delayed eosinophil apoptosis sustains tissue eosinophilia and recurrence.

### 2.6 Apoptosis in Neutrophils

Neutrophils resist apoptosis due to high Bcl‐2 levels, especially in refractory CRSwNP patients [103], where Bcl‐2 and HNE double‐positive cells suggest these proteins aid inflammatory cell survival and polyp recurrence [107]. Chronic inflammation caused by inhibited apoptosis is associated with granulocyte colony‐stimulating factor (G‐CSF) and IL‐6 [108]. G‐CSF is upregulated in patients with low‐symptom type 2 CRS [109], promoting neutrophil inflammation by reducing apoptosis in CRSwNP [110]. Meanwhile,IL‐6 reduces neutrophil recruitment and increases apoptosis in the innate immune response [111]; IL‐36 activates the NF‐κB pathway, producing IL‐6 and IL‐8. A study has shown reduced IL‐6 expression in CRSwNP [112]. GSK‐3 regulates inflammation and apoptosis, crucial for immune response, and is involved in IL‐6 signaling. In CRS, GSK‐3 expression increases but is phosphorylated and inhibited [113]. In clinical terms, neutrophil apoptosis resistance is more often discussed in non‐ECRS or Th2‐low phenotypes with neutrophil‐predominant inflammation.

### 2.7 Apoptosis in Olfactory Sensory Neurons and Olfactory Bulb Cells

Olfactory dysfunction (OD) is a common and clinically significant manifestation of CRS [114], particularly in severe disease phenotypes [115]. Accumulating evidence indicates that type 2 inflammation and eosinophilic infiltration within the olfactory mucosa act as key pathological drivers of OD aggravation rather than merely accompanying features [116]. These inflammatory changes are associated with mucosal erosion, neuroepithelial disruption, and loss of olfactory sensory neurons (OSNs), leading to impaired olfactory signal transduction [117].

Eosinophil‐derived cytotoxic granule proteins, including eosinophil cationic protein (ECP) and major basic protein (MBP), can directly induce OSN apoptosis, with neuronal loss correlating with the severity of olfactory impairment [117]. In parallel, persistent TNF‐α signaling in the olfactory mucosa promotes caspase‐dependent apoptosis of olfactory epithelial cells, thereby limiting neuronal regeneration and contributing to sustained OD in CRS [118, 119].

**FIGURE 3:**
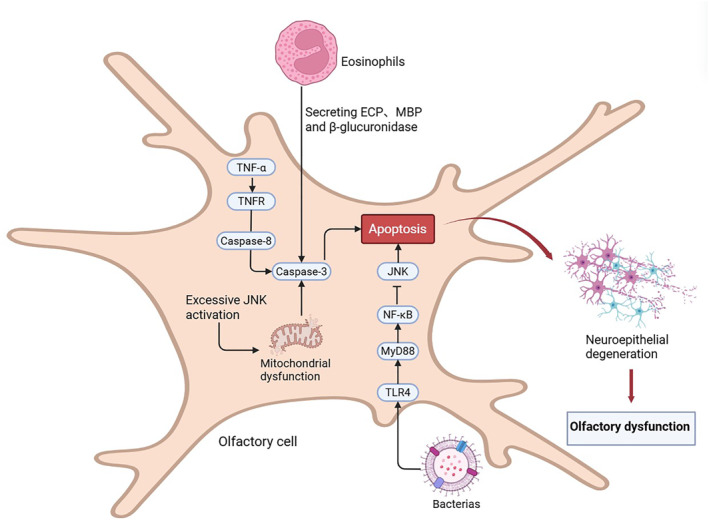
Apoptotic pathways underlying olfactory dysfunction in CRS. Chronic inflammation in the olfactory mucosa activates multiple apoptotic mechanisms in olfactory sensory neurons (OSNs). Eosinophil‐derived cytotoxic proteins (ECP, MBP, and β‐glucuronidase) and persistent TNF‐α signaling promote apoptosis through TNFR‐mediated caspase activation, culminating in caspase‐3–dependent cell death. Excessive JNK signaling induces mitochondrial dysfunction, further amplifying apoptotic signaling. Microbial stimuli activate the TLR4/MyD88/NF‐κB pathway, which may limit excessive JNK activation and modulate neuroinflammatory responses. These processes collectively lead to OSN apoptosis, neuroepithelial degeneration, and olfactory dysfunction.

Sustained activation of the JNK signaling pathway has also been implicated in inflammation‐induced OSN apoptosis, where JNK functions as a key mediator of apoptotic signaling [119, 120]. In contrast, NF‐κB signaling exerts a neuroprotective effect by restraining excessive JNK activation, potentially through the TLR4/MyD88/NF‐κB axis, which regulates neuroinflammatory responses in allergic airway disease and may similarly contribute to CRS‐associated OD [121, 122]. Limited evidence from animal models further suggests that chronic sinonasal inflammation may secondarily affect the olfactory bulb via sustained neuroinflammatory signaling (Figure 3).

## 3 Necroptosis

### 3.1 Necroptosis in Epithelial Cell

Necroptosis is a regulated form of inflammatory cell death mediated by the RIPK1–RIPK3–MLKL signaling cascade [123]. In CRSwNP tissues, necroptotic signaling is often characterized by dominant phosphorylation of MLKL (p‐MLKL), which is associated with the release of DAMPs such as IL‐1α and HMGB1 and correlates with increased levels of inflammatory cytokines and neutrophil infiltration. Macrophages appear to be a major cellular source of p‐MLKL in inflamed sinonasal mucosa [14].

Microbial colonization represents an important upstream trigger of necroptosis in CRS. *Staphylococcus aureus*, a key pathogen in CRS, can induce necroptotic cell death through several virulence factors [124]. The pore‐forming toxin α‐hemolysin disrupts epithelial membrane integrity and activates the RIPK1–RIPK3–MLKL signaling axis, leading to necroptosis and release of inflammatory mediators [125]. In addition, phenol‐soluble modulins (PSMs) and leukocidins such as LukAB and Panton–Valentine leukocidin (PVL) can induce necroptosis‐like inflammatory cell death in epithelial and immune cells [125]. These toxins trigger membrane damage and intracellular stress responses that converge on RIPK1/RIPK3 activation and MLKL phosphorylation.

**FIGURE 4:**
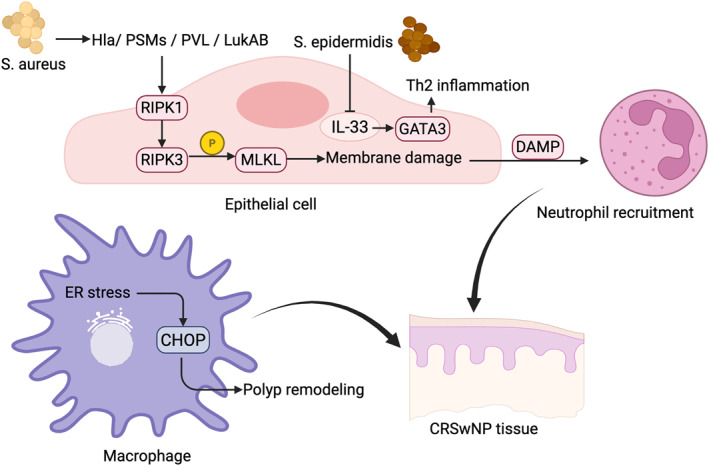
Molecular mechanisms of necroptosis in CRS. Pathogen‐induced epithelial necroptosis, ER stress–CHOP‐related macrophage remodeling, and the indirect IL‐33–Th2/GATA3 axis involved in necroptosis‐associated type 2 immune amplification in CRS.

Necroptotic cell death results in membrane rupture and the release of DAMPs, including HMGB1 and IL‐1α, which further amplify mucosal inflammation and recruit neutrophils [14]. In this way, S. aureus‐induced necroptosis may serve as an important mechanism linking microbial colonization to epithelial injury and persistent inflammation in CRS (Figure 4).

### 3.2 Necroptosis in Macrophages

Across airway diseases, macrophage ER stress–CHOP signaling is repeatedly linked to remodeling, but its direction is context dependent. In fibrosis and allergic airway inflammation models [126], CHOP has been reported to support M2 programs and profibrotic outputs, whereas in other fibrotic settings ER‐stress signaling can also promote macrophage apoptosis, thereby modulating macrophage accumulation and disease progression [127].

In CRSwNP, ER stress is activated and CHOP is preferentially enriched in macrophages; a subset of these cells undergoes apoptosis, whereas others show necroptosis features, with IL‐1β and ER stress enhancing this cell‐death landscape in polyp‐derived cell systems [32]. Meanwhile, M2‐skewed macrophage signals are repeatedly linked to polyp remodeling (e.g., growth‐factor and matrix programs) [31]. However, current data still do not resolve whether the increased M2 compartment reflects compensatory expansion of surviving macrophages or polarization driven by necroptosis‐derived DAMPs. Taken together, macrophage remodeling in CRSwNP likely represents an integrated readout of microenvironmental cues rather than a single linear CHOP→death→M2 axis (Figure 4).

### 3.3 Necroptosis‐Associated T‐Cell Polarization

In CRS, it is more coherent to frame “necroptosis–T cells” through an epithelium–alarmin–Th2 axis. Epithelial necroptosis can act as an inflammatory amplifier by boosting alarmin signaling, particularly IL‐33, thereby reinforcing Th2/GATA3‐skewed immunity. In contrast, *Staphylococcus* epidermidis reduces IL‐33 and GATA3 in HNECs and blunts allergen‐induced necroptosis, suggesting that it may dampen Th2 inflammation by lowering necroptosis‐linked alarmin output [34, 35].

This aligns with CRSwNP biology, where local type‐2 amplification is highly IL‐33 dependent, making microbiome control of IL‐33 and epithelial death programs a plausible node connecting colonization to T‐cell polarization [128]. A similar paradigm is reported in other airway settings, where epithelial necroptosis and IL‐33 release converge to amplify type‐2 inflammation, supporting cross‐airway relevance of this axis [14, 129]. Taken together, the most defensible interpretation is that epithelial necroptosis shapes the Th2/GATA3 milieu via IL‐33, while direct evidence for necroptosis occurring within T cells themselves remains limited (Figure 4).

## 4 Pyroptosis

**FIGURE 5:**
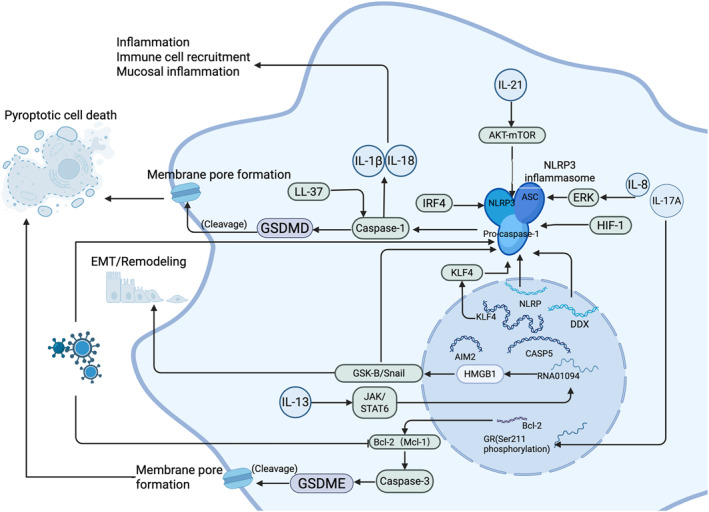
Pyroptosis signaling pathways in CRS. Multiple inflammatory signals converge on NLRP3 inflammasome activation in CRS. Activated NLRP3 promotes caspase‐1‐mediated GSDMD cleavage and IL‐1β/IL‐18 maturation, leading to membrane pore formation, pyroptotic cell death, and inflammatory amplification. A parallel caspase‐3/GSDME pathway is also involved as an alternative pyroptotic execution route. In addition, EMT is shown outside the cell to indicate its role as a downstream remodeling consequence rather than an intracellular execution step of pyroptosis.

Pyroptosis, a type of PCD triggered by inflammasome activation, exacerbates inflammation by releasing pro‐inflammatory cytokines [9]. The NLRP3 inflammasome, linked to various inflammatory diseases, consists of NLRP3, ASC, and pro‐caspase‐1 [130], promoting cytokine maturation like IL‐1β and IL‐18, leading to pyroptosis [131]. Dysfunction of the NLRP3 may significantly contribute to inflammatory diseases by exacerbating local inflammation [132] (Figure 5).

### 4.1 Pyroptosis in Epithelial Cells

NLRP3‐mediated pyroptosis is key to the nasal mucosal damage seen in patients with CRS [15]. Pyroptosis primarily occurs in the epithelial cells of the nasal cavity and sinuses in patients with CRS. CRS is closely associated with epithelial cell damage and inflammatory cell infiltration, both of which are related to the activation of pyroptosis. Research indicates that HIF‐1α enhances the stability of NLRP3, thereby preventing its degradation during hypoxia‐induced inflammation [38]. Researchers have identified key genes and proteins associated with pyroptosis in CRS through gene library studies, particularly in CRSwNP cases. Notable examples include the increased expression of AIM2, CASP5, and NLRP6 [40]. Notably, IL‐13 upregulates LINC01094 in CRSwNP‐derived nasal epithelial cells, and LINC01094 mediates IL‐13‐driven epithelial pyroptosis by upregulating HMGB1 and activating the downstream GSK‐3β/Snail signaling axis [37]. Pyroptosis worsens sinus inflammation by raising oxidative stress [9]. KLF4 is elevated in CRS nasal tissue, boosting NLRP3 expression, activating pyroptosis in NEpCs, leading to more sneezing, nose rubbing, and inflammatory cell infiltration [39]. NLRP3/caspase‐1/GSDMD‐N pathway initiates this process in allergic rhinitis (AR), while IRF4 worsens inflammation in CRSsNP [133], with IRF4 exacerbating inflammation in CRSsNP [134]. Research shows IL‐8 and IL‐17A induce pyroptosis in HNECs via the ERK‐NLRP3/caspase‐1 pathway, increasing IL‐1β and IL‐18 secretion [17, 36]. HMGB1 stimulates HNECs to produce IL‐6 and IL‐8 [37]. IL‐8 contributes to glucocorticoid resistance by influencing the phosphorylation of Ser211 on the glucocorticoid receptor, thereby disrupting receptor balance in patients with CRSwNP. Human nasal cells secrete IL‐1β and undergo pyroptosis via the DDX33/DDX58‐NLRP3‐caspase‐1‐GSDMD pathway when infected with hRV [41]. Research indicates that viruses inhibit Bcl‐2 proteins, such as Mcl‐1, which activates Gasdermin‐E(GSDME)‐dependent pyroptosis. This process leads to the death of airway epithelial cells, resembling findings in CRS [42]. In the ECRS mouse model, markers of pyroptosis in the nasal mucosa rise after viral infection [115], which may reverse the cell characteristics and result in cell death through pyroptosis.

### 4.2 Pyroptosis in Other Cells

Current research investigates proteins related to pyroptosis, such as LL‐37, which influences immune responses in the upper respiratory tract [135] and is found in CRS. Studies indicate LL‐37 causes cell death via caspase‐1 and ‐8 activation, resulting in human HNEC and macrophage death through necrosis or pyroptosis, not apoptosis [49]. Research has found that IL‐21 produced by T cells in CRS can induce pyroptosis in regulatory T cells (Treg) by activating the Akt‐mTOR‐NLRP3‐caspase‐1 signaling pathway [136]. Furthermore, pyroptosis is closely related to olfactory dysfunction and is primarily mediated by NLRP3 [41].

## 5 ETosis

**FIGURE 6:**
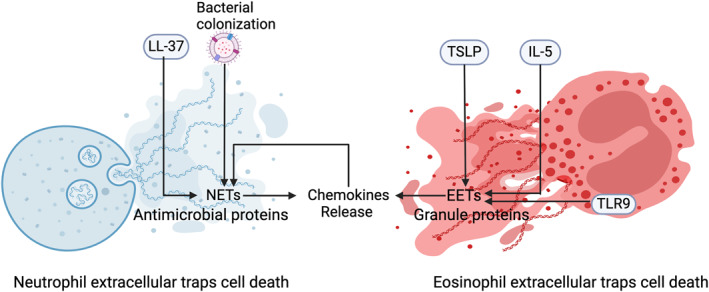
Molecular mechanisms of ETosis in CRS. LL‐37‐triggered NETosis, and TLR9‐mediated EETosis; Cross‐regulation: NETs and EETs form a chronic inflammatory loop by mutually enhancing chemokine secretion, perpetuating neutrophil/eosinophil recruitment and tissue damage in CRS.

Recent studies have introduced a new form of PCD known as ETosis. This process involves the release of ETs that are linked to immune issues in chronic inflammatory diseases [20], including chronic obstructive pulmonary disease (COPD), CRS, and asthma [137]. Immune cells like neutrophils and eosinophils use these extracellular traps for defense, particularly in CRS (Figure 6). Clinically, ETosis shows endotype skewing: eosinophil ETosis is more prominent in ECRS, whereas NET formation is more often reported in non‐ECRS CRSwNP.

### 5.1 Neutrophil Extracellular Trap Cell Death

NETs are extracellular structures of chromatin and proteins that bind and eliminate microorganisms [20]. Neutrophil stimulation causes nuclear shape change, membrane rupture, and NET release, differing from apoptosis [43] and necrosis due to reliance on ROS from NADPH oxidase [20]. The role of NETs in CRS remains debated. NETs are more common in patients with and linked to neutrophil infiltration in non‐eosinophilic CRSwNP,and LL‐37 triggers NETs in CRSwNP [45], with research indicating they may promote Ki‐67+ p63+ cell proliferation and are related to bacterial colonization [138]. Differences in eosinophil counts between refractory and non‐refractory groups suggest NET death could be a useful neutrophil marker in CRSwNP [139]. During exacerbations of CRS, the increase in NETs and neutrophils in nasal secretions results in the secretion of chemokines. This, in turn, enhances the epithelial barrier and promotes further neutrophil infiltration [140]. In severe CRSwNP, inflammatory factors IL‐1β and IL‐8 increase mucus hypersecretion and tissue remodeling, contributing to glucocorticoid resistance [141].

### 5.2 Eosinophil Extracellular Traps Cell Death

Eosinophil degranulation occurs when eosinophils release granules via ETosis, involving cell death and nuclear DNA traps [142]. Eosinophil ETosis in CRS captures pathogens via extracellular traps, enhancing inflammation and aiding infection resistance. This type of pro‐inflammatory cell death is associated with the cytokine IL‐5 and neutrophil activity linked to eosinophil trap death and Charcot‐Leyden crystals [46]. Li concluded EETs cause Th2 inflammation, damaging the mucosal barrier and promoting polyp formation through substances like IL‐5 and TSLP [47]. Yu demonstrated that cfDNA activates TLR9 signaling, triggering EET formation [48]. Gevaert's study shows eosinophils are recruited to *S*. *aureus* sites in CRS, forming traps to combat infections [143]. Excessive ETosis can worsen chronic inflammation; studies show eosinophils in CRS patients have significant ETosis activity linked to disease severity [140]. However, current research lacks in‐depth exploration of this mechanism, and we hope future studies will address it.

## 6 The Relationship Among Different Types of PCD

Accumulating evidence suggests subtype‐specific distributional preferences of programmed cell death pathways in CRS. Eosinophilic CRS is more closely associated with eosinophil apoptosis resistance and eosinophilic ETosis, whereas non‐eosinophilic CRS is characterized by neutrophilic inflammation, epithelial injury, and may involve enhanced pyroptosis and necroptosis. However, direct comparative evidence across CRS subtypes remains limited.

### 6.1 Apoptosis and Necroptosis

**FIGURE 7:**
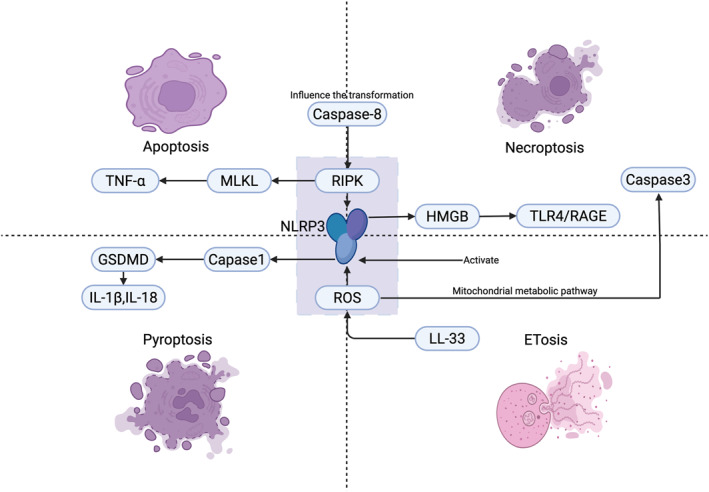
Molecular cross‐regulation of apoptosis, necroptosis, pyroptosis, and ETosis in CRS. ROS‐driven NLRP3 inflammasome activation triggered cell death; Apoptosis‐necroptosis switch regulated by caspase‐8.

Current research suggests that there is competition and conversion between apoptosis and necroptosis. Caspase‐8 regulates apoptosis, necroptosis, and ETosis, inhibiting RIPK1/RIPK3 to promote apoptosis [144]. Pathogens like *S*. *aureus* may enhance inflammation by promoting necroptosis via caspase inhibitors [33]. Viruses like influenza and RSV suppress anti‐apoptotic proteins Mcl‐1 and Bcl‐xL, activating the mitochondrial apoptotic pathway. They activate GSDME pyroptosis, causing airway epithelial cell death and barrier collapse [30]. This shows the competition between apoptosis and necroptosis. Insufficient apoptosis in CRS leads to necroptosis, increasing inflammation (Figure 7).

### 6.2 Pyroptosis and Apoptosis

Research also suggests that pyroptosis has a synergistic effect with apoptosis and necroptosis. ROS activate the NLRP3 inflammasome and damage mitochondria, promoting pyroptosis and apoptosis in CRS. Excessive ROS increases NLRP3 expression, activates caspase‐1, and raises pyroptosis marker GSDMD‐NT [145]. ROS also cause mitochondrial dysfunction, releasing cytochrome C to activate the caspase‐9/3 cascade for apoptosis [26]. GSDMD initiates pyroptosis and releases DAMPs [146], activating NF‐κB and NLRP3. Additionally, GSDMD pores in CRS cause mitochondrial damage and trigger apoptotic pathways like Caspase‐9 [145].

Research on CRS shows that smoke exposure causes IL‐1β and TNF‐α to work together, enhancing tissue remodeling and inflammation via RAGE signaling [24] (Figure 7).

### 6.3 Pyroptosis, Necroptosis and ETs

Research indicates PCD amplifies ETs, while Li reviews the mechanisms of ETs and recent studies on CRS [47]. Pyroptosis is associated with neutrophil infiltration and the formation of NETs [147]. Pyroptosis in HNECs correlates with NET levels, amplifying local inflammation. The GSDMD protein causes cell membrane perforation, releasing NETs similar to caspase‐11 driven NETosis [148]. When macrophages phagocytose NETs, they activate inflammasomes such as NLRP3, which exacerbates lung injury, creating a vicious cycle. Research also shows that ROS is linked to pyroptosis and reducing ROS can inhibit NET formation [44]. Research on EETs is limited. Necrosis releases ATP, while autophagy reduces ATP, inhibiting EETs in asthma patients [149]. A similar mechanism may exist in CRS, warranting further study. Research shows that the RIPK3‐MLKL pathway is involved in necroptosis and sometimes helps release NETs [150]. However, XU questions if necrotic cell death and released DAMPs recruit neutrophils in CRSwNP [14] (Figure 7).

### 6.4 PANoptosis and PCD

PANoptosis has been proposed as an integrated cell‐death program that co‐engages hallmarks of apoptosis, necroptosis, and pyroptosis, organized by multifaceted PANoptosome complexes [151]. At the mechanistic level, PANoptosome signaling can be initiated by TNF‐α and IFN‐γ, leading to caspase‐8 activation and coordinated execution of pyroptotic, apoptotic, and necroptotic modules [152]. This convergence culminates in PANoptosis and has been linked to exacerbated tissue damage [153, 154].

In the respiratory tract, several prototypical settings support these concepts. TNF‐α/IFN‐γ synergy can drive a caspase‐8–centered inflammatory death program with coupled RIPK‐ and inflammasome‐linked pathways, and this response is associated with worsened injury [154]. In viral pneumonia, the ZBP1‐PANoptosome has been invoked to explain concurrent activation of multiple death pathways during influenza infection [155]. More recently, PANoptosis has also been implicated in SARS‐CoV‐2–related bystander‐cell pathology [156].

In CRS, multiple PCD signatures often overlap across epithelial and immune compartments, and PANoptosis provides a useful interpretive lens for this redundancy in chronic sinonasal inflammation [151]. Direct evidence for PANoptosome assembly in CRS is still limited. Accordingly, PANoptosis is best positioned as a working concept that encourages time‐matched, multi‐axis profiling of apoptotic, necroptotic, and pyroptotic nodes alongside upstream inflammatory drivers, rather than assigning the phenotype to a single pathway [157].

## 7 Potential Therapeutic Targets

A substantial proportion of the pharmacologic evidence summarized below is derived from asthma, AR, or respiratory disease models rather than CRS‐specific interventional studies. We specify disease sources and interpret data as support for targeting cell‐death pathways in airway inflammation, awaiting CRS validation.

### 7.1 Potential Therapeutic Targets of Apoptosis

#### 7.1.1 Activating the JNK/MAPK Pathway

Activation of the JNK/MAPK pathway promotes apoptosis. Pepsin activates JNK/MAPK, increasing HSP70 and HNEpC apoptosis [158]. Quercetin inhibits XBP1‐induced apoptosis [57] and reverses its inhibition of HNEC proliferation and HIF‐1α/Wnt‐β‐catenin activation^160.^. Dexamethasone induces HNEC apoptosis, while PDT triggers apoptosis in epithelial cells and leukocytes to control inflammation [159]. MTX triggers apoptosis in NP cells through caspase cascades, mitochondrial pathways, and the p38 MAPK/JNK pathway [160]. However, recent studies indicate that using p38 MAPK inhibitors can decrease cell death rates in OSNs and significantly enhance olfactory function in AR mice [161] which may provide mechanistic insight but is not CRS‐specific evidence (Table 2).

**TABLE 2:** Potential therapeutic targets in CRS.

PCD type	Therapeutic mechanism	Treatments (drugs)	Effects
Apoptosis	Activating JNK/MAPK	Pepsin	Pepsin activates JNK/MAPK and induces HNEpC apoptosis [158].
	Inhibiting XBP1/ER‐stress apoptosis	Quercetin	Quercetin inhibits XBP1‐driven epithelial apoptosis under ER stress [57, 162].
	Inducing apoptosis in inflammatory cells	MTX	MTX induces apoptosis in nasal polyp cells and reduces polyp burden [160].
	Inducing apoptosis (glucocorticoid/PDT)	Dexamethasone; PDT	Dexamethasone and PDT induce apoptosis in epithelial and inflammatory cells in CRS models [159].
	Inhibiting p38 MAPK/JNK (OD protection)	SB203580	[Non‐CRS: Allergic rhinitis model] SB203580 inhibits p38 MAPK, reduces OSN death, and improves olfactory function [161].
	Inhibiting BCL‐2 family (pro‐apoptotic)	Venetoclax; ABT‐737; ABT‐199	[Non‐CRS: Asthma/airway models] BCL‐2 inhibition reduces eosinophil infiltration and airway hyperreactivity [163]. [Non‐CRS: Airway models] ABT‐737/ABT‐199 induce neutrophil apoptosis and alleviate corticosteroid‐resistant inflammation [164, 165].
	Inducing fibroblast apoptosis (anti‐fibrotic)	BLE‐A5	BLE‐A5 induces nasal polyp fibroblast apoptosis and inhibits ECM deposition [166, 167].
	Blocking IL‐4/IL‐13 axis (restore apoptosis balance)	Dupilumab; Luteolin	Dupilumab inhibits IL‐4/IL‐13 signaling and improves ECRS outcomes. Luteolin downregulates BCL‐2‐associated anti‐apoptotic signaling and promotes apoptosis rebalancing [168].
	Blocking caspase‐1/2/3 activity	Z‐VAD‐FMK; Ac‐YVAD‐cmk; Quercetin	[Non‐CRS: Airway models] Z‐VAD‐FMK reduces epithelial apoptosis [169]. Ac‐YVAD‐cmk and Z‐VAD‐FMK reduce olfactory epithelial apoptosis in CRS‐related OD settings [117]. [Non‐CRS: Allergic rhinitis model] Quercetin lowers caspase‐3 expression [170].
	Mitochondrial stabilization/lysosomal apoptosis	Glutathione; Siglec‐8; Saponin	Glutathione stabilizes mitochondrial function and limits apoptosis‐associated remodeling [95]. Siglec‐8 activates lysosomal apoptosis in eosinophils/mast cells [171].82,175 Saponin induces eosinophil and HMC‐1.2 cell death [171].
	Steroids and apoptosis regulation	Corticosteroids	Corticosteroids promote apoptosis of ILC2 and reduce type 2 inflammation [172]. Local corticosteroids may induce neuronal apoptosis and contribute to olfactory impairment [173].
Pyroptosis	Blocking NLRP3 inflammasome	MCC950; Glycyrrhizin	[Non‐CRS: Allergic rhinitis model] MCC950 inhibits NLRP3 and reduces caspase‐1/IL‐1β/IL‐18 [174, 175]. [Non‐CRS: Asthma models] Glycyrrhizin blocks NLRP3 inflammasome assembly and improves airway remodeling [176].
	Inhibiting NLRP3 activation/suppressing ER stress	Platycodon D; Xiao Qing Long Decoction; Su Huang	Platycodon D activates Nrf2/HO‐1, reduces ROS, and inhibits NLRP3 pyroptosis in HNECs [145]. [Non‐CRS: Airway models] Xiao Qing Long Decoction suppresses pyroptosis‐related inflammation [177]. [Non‐CRS: cough variant asthma model] Su Huang disrupts NLRP3 assembly and reduces caspase‐1/IL‐1β [178].
	Inhibiting GSDMD (pore formation)	GSDMD inhibitors; Heme oxygenase‐1 (HO‐1)	GSDMD inhibitors block pore formation and IL‐1β release.17 HO‐1 suppresses NF‐κB signaling and limits GSDMD‐N–mediated pyroptosis [179, 180].
	Blocking TLR4/MyD88/NF‐κB pathway	MUC1; Protopine; PVP‐I	[Non‐CRS: Asthma model] MUC1 inhibits TLR4/MyD88/NF‐κB and reduces airway inflammation [181]. [Non‐CRS: OVA‐asthma model] Protopine alleviates airway inflammation via NF‐κB inhibition [182]. PVP‐I blocks NF‐κB nuclear translocation in non‐eosinophilic CRS and reduces caspase‐1 pyroptosis and IL‐1β release [183].
	Blocking HMGB1–TLR4 signaling	TLR4‐blocking antibody	TLR4‐blocking antibodies reduce HMGB1‐induced IL‐6/IL‐8 secretion and suppress pyroptosis‐associated inflammation [37].
	HMGB1 antagonism (systemic evidence)	Glycyrrhizin	[Non‐CRS: Rhinitis] Glycyrrhizin reduces HMGB1 levels and induces eosinophil death [184].
Necroptosis	Blocking RIPK1/RIPK3/MLKL pathway	Torin‐1; SMZCL	[Non‐CRS: Asthma] Torin‐1 inhibits mTOR and reduces RIPK3/MLKL phosphorylation; SMZCL targets this pathway [185].
	Blocking RIPK3/MLKL (CRS‐relevant trigger)	RIPK3 inhibitor; MLKL inhibitor	TNF‐α + IFN‐γ induce RIPK3–MLKL necroptosis in CRS‐related THP‐1 models, and RIPK3/MLKL inhibitors block this process [14].
	Blocking RIPK1	Nec‐1	Nec‐1 inhibits RIPK1 kinase activity and prevents necrosome formation [186]. [Non‐CRS: Airway models] Nec‐1 mitigates TNF‐α–induced necroptosis associated with MUC1 downregulation and glucocorticoid resistance [187]. Nec‐1 inhibits MLKL phosphorylation and membrane perforation [188].
	Biologic therapy (IL‐33/ST2 axis)	Astegolimab	[Non‐CRS: Asthma trials] Astegolimab blocks ST2 and modulates Th2 immunity; CRS‐specific clinical evidence is lacking [189].
ETosis	Inhibiting PAD4 (histone citrullination)	PAD4 inhibitors; simvastatin; CIT‐013	PAD4 inhibitors block histone citrullination and ET formation [190, 191]. [Non‐CRS: Lower‐airway disease models] PAD4 inhibition reduces NETs and airway inflammation [192]. [Non‐CRS: Severe asthma] simvastatin lowers PAD4 and inhibits NETosis [193]. CIT‐013 blocks EET release by targeting citrullinated histones [194].
	Blocking IL‐33–driven NETosis amplification	IL‐33 blockade	[Non‐CRS: Asthma] IL‐33 blockade reduces neutrophilic inflammation and NETosis [195].
	Blocking neutrophil elastase/histone cleavage	Sivelestat; AAT infusion	Sivelestat inhibits histone cleavage signaling and reduces polyp formation [138]. [Non‐CRS: AATD/chronic respiratory disorders] AAT inhibits neutrophil elastase and can reduce NETs; AAT infusion slows emphysema/asthma progression in AATD [196, 197, 198].
	Removing ROS (antioxidant strategies)	Nrf2/HO‐1 activation; antioxidants	Nrf2/HO‐1 activation reduces ROS and indirectly limits ETosis [145]. ROS reduction attenuates PAD4 activation and limits NET/EET release [190, 191, 192]. [Non‐CRS/limited CRS evidence] targeting oxidative stress may improve severe airway inflammation and steroid resistance [44, 193].
	Degrading formed ETs	DNase I (rhDNase); Pulmozyme; TiS_2_ nanosheets	[Non‐CRS: Asthma] DNase I reduces airway hyperreactivity and inflammation by degrading NETs [199]. [Non‐CRS: Cystic fibrosis] Inhaled rhDNase improves FEV1 and reduces sputum viscosity; effects may vary across trials [200, 201]. [Non‐CRS: Chronic lung disease] Pulmozyme is associated with side effects [202]. DNase I nasal spray decreases IL‐1β levels in CRS [138]. TiS_2_ nanosheets deliver DNase I for CRS treatment [48].
	Stopping downstream ET‐DNA signaling	CpG oligodeoxynucleotides (TLR9 agonists)	[Non‐CRS: Allergic respiratory disease/COPD models] CpG ODNs block NET‐DNA–TLR9 signaling, inhibit NF‐κB, and reduce IL‐6/IL‐8 [203, 204].
	Blocking NET‐induced IL‐1β signaling	Canakinumab	[Non‐CRS: Asthma] Canakinumab blocks IL‐1β signaling induced by NETs [205].
	Blocking CXCR2/IL‐8 axis	CXCR2 antagonist (AZD5069)	[Non‐CRS: Asthma/bronchiectasis trials] CXCR2 blockade reduces neutrophil recruitment and may limit NET formation; CRS evidence is lacking [206, 207].

#### 7.1.2 Modulating Proteins Involved in Apoptosis

Bcl‐2 is key treatment target. Venetoclax reduces eosinophil infiltration and airway hyperreactivity in asthma. It is also included in treatment‐related patents [163]. Although CRS and asthma share the unified airway concept, their local microenvironments differ substantially [208]. Therefore, asthma‐derived therapeutic evidence (e.g., Venetoclax) should be interpreted cautiously when applied to CRS.

pH‐sensitive nanomedicines of Bcl‐2 inhibitors ABT‐737 or ABT‐199 effectively treat eosinophilic and neutrophilic airway inflammation and can induce neutrophil apoptosis [164], alleviating corticosteroid‐resistant inflammation [165]. However, these studies are largely from non‐CRS airway disease models. BLE‐A5 induces pro‐apoptotic effects in fibroblasts derived from NP by regulating Bcl‐2 activity and activating caspases [166], Additionally, it inhibits Drp1 phosphorylation, which disrupts mitochondrial dynamics and triggers cell death [167].

Some antibodies influence Bcl‐2 expression. Dupilumab is a monoclonal antibody that targets the IL‐4 receptor α. It blocks IL‐4/IL‐13 signaling, suppresses STAT6, reduces eosinophil survival, and decreases Bcl‐2 expression. In the ECRS model, reduced Bcl‐2 expression leads to olfactory neuron death, while luteolin boosts Bcl‐2 via TLR4/NF‐κB modulation, decreasing apoptosis proteins and enhancing smell [168].

#### 7.1.3 Blocking the Caspase Activity

Caspase enzymes are key targets in cell death mechanisms. Z‐VAD‐FMK reduces apoptosis in airway epithelial cells [169] (non‐CRS airway models), while Ac‐YVAD‐cmk selectively inhibits caspase‐1. Both inhibit caspases 1, 2, and 3 involved in TNF‐α‐induced cell death in olfactory epithelial cells [117]. Quercetin reduces inflammation by regulating cytokines and epithelial cell death, lowering caspase‐3 in AR mice [170].

#### 7.1.4 Other Treatments

Mechanistic studies emphasize the critical role of the mitochondrial pathway in cellular processes. Glutathione prevents the degradation of the ECM and apoptosis by stabilizing the mitochondrial membrane, thereby suggesting new clinical targets for intervention [95]. Siglec‐8 triggers lysosomal apoptosis [171], and saponin significantly kills eosinophils and HMC‐1.2 cells, offering new therapeutic targets. Corticosteroids reduce eosinophilia by promoting apoptosis in ILC2 [172]. Meanwhile, MP analysis may identify biomarkers for personalized treatment [52], indicating a multifaceted approach to ECRS. Furthermore, research indicates that local corticosteroids can impair olfactory function by causing neuronal apoptosis and disrupting the OSN renewal process [173].

### 7.2 Potential Therapeutic Targets of Pyroptosis

#### 7.2.1 Blocking the NLRP3 Inflammasome

Targeted therapy for pyroptosis shows promise in reducing CRS inflammation by inhibiting. MCC950, a selective NLRP3 inhibitor [174], reduces Caspase‐1, ASC, IL‐1β, and IL‐18 secretion and nasal damage in AR mice [175]. Natural compounds, such as glycyrrhizin, also block the assembly of the NLRP3 inflammasome and enhance airway remodeling in mice suffering from asthma [176]. These non‐CRS studies support mechanistic plausibility, but CRS‐specific interventional evidence remains limited.

#### 7.2.2 Blocking the Activation of the NLRP3 Inflammasome

Certain traditional Chinese medicines have been shown to inhibit the activation of the NLRP3. Platycodon D (PD) from Platycodon grandiflorus activates the Nrf2/HO‐1 pathway to reduce ROS, blocking NLRP3 and pyroptosis in HNECs [145]. Xiao Qing Long Decoction suppresses pyroptosis in nasal mucosal cells [177], while Su Huang disrupts NLRP3 assembly and lowers caspase‐1 expression, decreasing IL‐1β secretion [178].

#### 7.2.3 Inhibition of Gasdermin D

In CRS, Inhibition of Gasdermin D (GSDMD) inhibitors block pore formation and the release of IL‐1β [17]. Antibodies that target the N‐terminus of GSDMD prevent pyroptosis. Heme oxygenase‐1 inhibits GSDMD‐mediated pyroptosis and reduces TSLP release by binding to NF‐κB^181^, also limiting GSDMD N‐terminus release and affecting NLRP3‐caspase 1‐ GSDMD trimer formation [180]. Nonylphenol exposure triggers NLRP3 inflammasome and GSDMD‐mediated pyroptosis in the nasal mucosa [181].

#### 7.2.4 Blocking the TLR4/MyD88/NF‐κB Signaling Pathway

Inhibiting the TLR4/MyD88/NF‐κB pathway alleviates NLRP3 pyroptosis; MUC1 can therefore reduce airway inflammation in asthma [181] and Protopine eases OVA‐induced asthma [182], while PVP‐I blocks NF‐κB movement in non‐eosinophilic CRS, reducing CASPASE‐1 pyroptosis and IL‐1β release, thus lowering mucous membrane inflammation [183].

HMGB1, a damage‐associated molecular patterns(DAMPs), activates immune receptors like TLR4 and RAGE, enhancing inflammation [209]. The combination of glycyrrhizin and HMGB1 reduces HMGB1 levels in rhinitis patients and induces eosinophil death [184] (non‐CRS evidence). Overexpressed HDAC4 prevents HMGB1‐induced HNEC pyroptosis, while Sphk1 regulates HMGB1 through HDAC4, influencing epithelial cell pyroptosis in AR [210]. Also the study found that antibodies blocking Toll‐like receptor 4 reduced IL‐6 and IL‐8 secretion induced by HMGB1, preventing pyroptosis [37].

### 7.3 Potential Therapeutic Targets of Necroptosis

#### 7.3.1 Blocking the RIPK1/RIPK3/MLKL Pathway

Inhibiting mTOR with Torin‐1 reduces RIPK3 and MLKL phosphorylation, leading to cell death, and the Chinese medicine formula SMZCL targets asthma's necroptotic pathway by this [185] (non‐CRS evidence). Research indicates that TNF‐α and IFN‐γ in CRS activate the RIPK3‐MLKL pathways, leading to necroptosis in THP‐1 cells. Both RIPK3 and MLKL inhibitors can effectively block this process [14]. Blocking RIPK1 reduces necroptosis in respiratory diseases and improves barrier function [211]. Necrostatin‐1 inhibits RIPK1 kinase activity, preventing necrosome formation [186]. MUC1 is a mucin glycoprotein on respiratory epithelial cells; its downregulation weakens glucocorticoid effects against necroptosis [187] and affects the RIPK1/RIPK3 pathway, increasing TNF‐α induced necroptosis, though Nec‐1 can mitigate this [187]. Current research shows Nec‐1 inhibits MLKL phosphorylation and perforation [188], but MLKL inhibitors in CRS are under‐researched.

#### 7.3.2 Other Types of Biological Preparations

Certain biologic therapies are known to inhibit Th2 inflammation and necroptotic cell death. Astegolimab is a human IgG 2 monoclonal antibody that inhibits the IL‐33 receptor ST2, regulating type 2 immune responses and effective in asthma trials [189]. However, CRS‐specific clinical evidence is currently lacking.

### 7.4 Potential Therapeutic Targets of ETosis

#### 7.4.1 Inhibit the Formation of ETosis

To inhibit the formation of ETosis, PAD4 converts arginine in histones H3/H4 to citrulline, unwinding chromatin and forming ETs [190]. PAD4 inhibitors block this by binding to the enzyme's active site [191]. Inhibiting PAD4 expression can reduce NET formation and lower airway inflammation scores [192], although much of this evidence is derived from non‐CRS lower‐airway disease models. Simvastatin reduces Th17‐driven neutrophil inflammation and airway hyperreactivity by lowering PAD4 and inhibiting NETosis in severe asthma mice [193] (non‐CRS evidence). CIT‐013, a monoclonal antibody targeting citrullinated histones H2A and H4, effectively inhibits EET release without impacting eosinophil functions [194]. Inhibiting inflammatory mediators can prevent NETs in lung diseases like asthma [212], where blocking IL‐33 may reduce asthma severity [195]. The chemokine receptor Additionally, some protein inhibitors can also affect the formation of NETs. Sivelestat inhibits ΔNp63+ epithelial stem cell overexpression and polyp formation by blocking histone cleavage and signaling proteins [138]. Alpha‐1 antitrypsin deficiency (AATD) is a genetic condition associated with chronic respiratory issues like asthma and bronchiectasis [196]. AAT inhibits neutrophil elastase, which helps prevent NETs [197]. Currently, infusing AAT is the main treatment for AATD, slowing emphysema and asthma progression [198].

#### 7.4.2 Remove the ROS

Reactive oxygen species (ROS) are essential drivers of ETosis in CRS. Both neutrophil and eosinophil extracellular trap formation rely on ROS generated mainly through NADPH oxidase activity. Excessive ROS not only promotes chromatin decondensation and extracellular trap release but also amplifies local inflammation by activating the NLRP3 inflammasome and inducing mitochondrial dysfunction, thereby linking ETosis with pyroptosis and apoptosis [44, 145].

Reducing intracellular ROS has therefore emerged as a potential strategy to limit ETosis‐driven inflammation. Activation of the Nrf2/HO‐1 antioxidant pathway suppresses ROS accumulation and inhibits NLRP3 inflammasome activation in nasal epithelial cells, indirectly reducing extracellular trap formation [145]. In parallel, ROS reduction attenuates PAD4 activation, a key enzyme mediating histone citrullination and chromatin relaxation during ETosis, thereby limiting NET and EET release [190, 191, 192].

Although direct clinical evidence in CRS remains limited, experimental studies suggest that targeting oxidative stress may alleviate mucosal inflammation, epithelial barrier disruption, and steroid resistance associated with severe CRS phenotypes [44, 193].

#### 7.4.3 Break Down the Formed ETs

Breaking down ETs is a current research hotspot; DNase I hydrolyzes DNA bonds, disrupting ET structure. DNase I reduces airway hyperreactivity and inflammation by breaking down NETs [199]. Inhaled rhDNase improves FEV1 and reduces sputum viscosity [200], but Mayer believes there are no significant lung function changes after 6 weeks of treatment [201]. Likewise, Tarrant noted that Pulmozyme, similar to rhDNase, is associated with several side effects [202]. The application of DNase I spray in patients with CRS decreases IL‐1β levels [138]. Additionally, TiS_2_ nanosheets effectively capture DNase I for the treatment of CRS [48].

#### 7.4.4 Stop the Downstream Signals From ETs

CpG oligodeoxynucleotides, as TLR9 agonists, can prevent allergic respiratory diseases by blocking NETs‐DNA binding to TLR9, inhibiting the NF‐κB pathway and reducing IL‐6 and IL‐8 release [203, 204]. Canakinumab is a monoclonal antibody that inhibits IL‐1β signaling induced by NETs and is currently used for asthma treatment [205]. CXCR2 aids neutrophil movement to airways and induces NET formation via IL‐8 [206]. CXCR2 blockers are mostly studied for bronchodilation and asthma [207]. However, they haven't really been applied in CRS either.

## 8 Limitation

Despite significant progress in recent years concerning the association between CRS and PCD, the current findings exhibit several key limitations that hinder their clinical application. Current research on CRS has shown a significant gap in the in vitro validation of molecular mechanisms. Current research emphasizes animal models and tissue proteins, neglecting specific cell types in like eosinophils, fibroblasts, and epithelial cells. For instance, the timing and location of apoptosis, necroptosis, and pyroptosis proteins (the Caspase family, RIPK1/3, and GSDMD) in CRS cell subpopulations are unclear. Furthermore, the absence of in vitro experiments utilizing human primary cells, including gene knockout or overexpression studies and drug interventions, poses a significant barrier to effectively analyzing the key signaling pathways involved in CRS. Moreover, the clinical use of targeted therapies is falling behind. Although targeting PCD pathways (inhibiting NLRP3 inflammasome or blocking BCL‐2) has shown anti‐inflammatory potential in animal models, they face several challenges in clinical use. CRS patients show significant subtype differences, and a single target may only work for some. Systemic PCD inhibition may disrupt cell turnover (mucosal repair), risking infection or fibrosis. Local nasal treatments face issues such as poor drug stability and low mucosal penetration, limiting their effectiveness.

## 9 Conclusion

PCD plays a key role in CRS pathogenesis by regulating cell survival and influencing inflammation and immunity. Understanding PCD mechanisms can reveal CRS pathophysiology and lead to new treatment strategies, enhancing personalized care for patients. In summary, multiple forms of programmed cell death contribute to epithelial injury and chronic inflammation in CRS, and their interactions should be considered when interpreting disease mechanisms and therapeutic targets. Challenges in the single death pathway paradigm in CRS reveals new molecular mechanisms and supports multi‐target therapies for precise treatment.

## Author Contributions

**Xingchen Liu:** conceptualization, writing – original draft, data curation, methodology. **Junying Hu:** data curation, writing – original draft, writing – review and editing. **Weigang Gan:** writing – review and editing. **Qi Wu:** writing – review and editing. **Bing Zhong:** supervision, writing – review and editing. **Feng Liu:** funding acquisition, supervision, writing – review and editing.

## Funding

This work was supported by the National Natural Science Foundation of China (Grant 82401328) and the SCI Research Fund of West China Hospital, Sichuan University (Grant 141110062), awarded to Feng Liu. The funding body had no role in the design of the study, data interpretation, or writing of the manuscript.

## Ethics Statement

The authors have nothing to report.

## Conflicts of Interest

The authors declare no conflicts of interest.

## Data Availability

No new datasets were generated or analyzed during this study. Data sharing is therefore not applicable to this article.

## References

[1] W. J. Fokkens , V. J. Lund , C. Hopkins , et al., “European Position Paper on Rhinosinusitis and Nasal Polyps 2020,” Rhin (2020): 1–464, 10.4193/rhin20.600. 32077450

[2] T. Hildenbrand , K. Milger‐Kneidinger , I. Baumann , and R. Weber , The Diagnosis and Treatment of Chronic Rhinosinusitis (Deutsches Ärzteblatt international, 2024). 10.3238/arztebl.m2024.0167 PMC11741551 39173076

[3] R. R. Orlandi , T. T. Kingdom , T. L. Smith , et al., “International Consensus Statement on Allergy and Rhinology: Rhinosinusitis 2021,” International Forum of Allergy & Rhinology 11, no. 3 (2021): 213–739, 10.1002/alr.22741. 33236525

[4] C. Bachert , J. K. Han , M. Wagenmann , et al., “EUFOREA Expert Board Meeting on Uncontrolled Severe Chronic Rhinosinusitis With Nasal Polyps (CRSwNP) and Biologics: Definitions and Management,” Journal of Allergy and Clinical Immunology 147, no. 1 (2021): 29–36, 10.1016/j.jaci.2020.11.013. 33227318

[5] S. C. Payne , J. K. Han , P. Huyett , et al., “Microarray Analysis of Distinct Gene Transcription Profiles in Non‐Eosinophilic Chronic Sinusitis With Nasal Polyps,” American Journal of Rhinology 22, no. 6 (2008): 568–581, 10.2500/ajr.2008.22.3233. 19178793 PMC2810097

[6] S. Bedoui , M. J. Herold , and A. Strasser , “Emerging Connectivity of Programmed Cell Death Pathways and Its Physiological Implications,” Nature Reviews Molecular Cell Biology 21, no. 11 (2020): 678–695, 10.1038/s41580-020-0270-8. 32873928

[7] J. Yuan and D. Ofengeim , “A Guide to Cell Death Pathways,” Nature Reviews Molecular Cell Biology 25, no. 5 (2024): 379–395, 10.1038/s41580-023-00689-6. 38110635

[8] A. W. Griffioen and P. Nowak‐Sliwinska , “Programmed Cell Death Lives,” Apoptosis 27, no. 9–10 (2022): 619–621, 10.1007/s10495-022-01758-5. 35943678 PMC9361233

[9] K. Newton , A. Strasser , N. Kayagaki , and V. M. Dixit , “Cell Death,” Cell 187, no. 2 (2024): 235–256, 10.1016/j.cell.2023.11.044. 38242081

[10] S. Christgen , R. E. Tweedell , and T. D. Kanneganti , “Programming Inflammatory Cell Death for Therapy,” Pharmacology & Therapeutics 232 (2022): 108010, 10.1016/j.pharmthera.2021.108010. 34619283 PMC8930427

[11] W. Kiess and B. Gallaher , “Hormonal Control of Programmed Cell Death/Apoptosis,” European Journal of Endocrinology (1998): 482–491, 10.1530/eje.0.1380482. 9625357

[12] S. Kowalski , J. Karska , Z. Łapińska , B. Hetnał , J. Saczko , and J. Kulbacka , “An Overview of Programmed Cell Death: Apoptosis and pyroptosis—Mechanisms, Differences, and Significance in Organism Physiology and Pathophysiology,” Journal of Cellular Biochemistry 124, no. 6 (2023): 765–784, 10.1002/jcb.30413. 37269535

[13] J. Yan , P. Wan , S. Choksi , and Z. G. Liu , “Necroptosis and Tumor Progression,” Trends in Cancer 8, no. 1 (2022): 21–27, 10.1016/j.trecan.2021.09.003. 34627742 PMC8702466

[14] Y. Xie , M. Li , K. Chen , et al., “Necroptosis Underlies Neutrophilic Inflammation Associated With the Chronic Rhinosinusitis With Nasal Polyps (CRSwNP),” JIR 14 (2021): 3969–3983, 10.2147/jir.s322875. PMC8380290 34429629

[15] S. O. Vasudevan , B. Behl , and V. A. Rathinam , “Pyroptosis‐Induced Inflammation and Tissue Damage,” Seminars in Immunology 69 (2023): 101781, 10.1016/j.smim.2023.101781. 37352727 PMC10598759

[16] Z. Rao , Y. Zhu , P. Yang , et al., “Pyroptosis in Inflammatory Diseases and Cancer,” Theranostics 12, no. 9 (2022): 4310–4329, 10.7150/thno.71086. 35673561 PMC9169370

[17] Y. Li , L. H. Chang , W. Q. Huang , et al., “IL‐17A Mediates Pyroptosis via the ERK Pathway and Contributes to Steroid Resistance in CRSwNP,” Journal of Allergy and Clinical Immunology 150, no. 2 (2022): 337–351, 10.1016/j.jaci.2022.02.031. 35346673

[18] R. J. Nija , S. Sanju , N. Sidharthan , and U. Mony , “Extracellular Trap by Blood Cells: Clinical Implications,” Tissue Engineering and Regenerative Medicine 17, no. 2 (2020): 141–153, 10.1007/s13770-020-00241-z. 32114678 PMC7105514

[19] S. Ueki , Y. Konno , M. Takeda , et al., “Eosinophil Extracellular Trap Cell Death–Derived DNA Traps: Their Presence in Secretions and Functional Attributes,” Journal of Allergy and Clinical Immunology 137, no. 1 (2016): 258–267, 10.1016/j.jaci.2015.04.041. 26070883 PMC4674385

[20] T. A. Fuchs , U. Abed , C. Goosmann , et al., “Novel Cell Death Program Leads to Neutrophil Extracellular Traps,” Journal of Cell Biology 176, no. 2 (2007): 231–241, 10.1083/jcb.200606027. 17210947 PMC2063942

[21] T. M. Basinski , D. Holzmann , T. Eiwegger , et al., “Dual Nature of T Cell–Epithelium Interaction in Chronic Rhinosinusitis,” Journal of Allergy and Clinical Immunology 124, no. 1 (2009): 74–80.e8, 10.1016/j.jaci.2009.04.019. 19523671

[22] H. Lin , D. Lin , and X. Xiong , “Differential Expression of Livin, Caspase‐3, and Second Mitochondria‐Derived Activator of Caspases in Chronic Rhinosinusitis With Nasal Polyps,” Otolaryngol—Head neck surg. 151, no. 6 (2014): 1067–1072, 10.1177/0194599814551142. 25238746

[23] B. Wang , P. Cao , Z. Wang , et al., “Interferon‐γ‐Induced Insufficient Autophagy Contributes to p62‐Dependent Apoptosis of Epithelial Cells in Chronic Rhinosinusitis With Nasal Polyps,” Allergy 72, no. 9 (2017): 1384–1397, 10.1111/all.13153. 28258963

[24] H. Robin , C. Trudeau , A. Robbins , et al., “A Potential Role for the Receptor for Advanced Glycation End‐Products (RAGE) in the Development of Secondhand Smoke‐Induced Chronic Sinusitis,” Cimbebasia 46, no. 1 (2024): 729–740, 10.3390/cimb46010047. PMC10814859 38248349

[25] M. Jia , X. Chen , J. Liu , and J. Chen , “PTEN Promotes Apoptosis of H2O2‐Injured Rat Nasal Epithelial Cells Through PI3K/Akt and Other Pathways,” Molecular Medicine Reports (2017), 10.3892/mmr.2017.7912. 29115519

[26] H. Hu , F. Zhang , Z. Sheng , et al., “Synthesis and Mitochondria‐Localized Iridium (III) Complexes Induce Cell Death Through Pyroptosis and Ferroptosis Pathways,” European Journal of Medicinal Chemistry 268 (2024): 116295, 10.1016/j.ejmech.2024.116295. 38437750

[27] X. Xie , L. Xuan , Y. Zhao , X. Wang , and L. Zhang , “Diverse Endotypes of Chronic Rhinosinusitis and Clinical Implications,” Clinical Reviews in Allergy and Immunology 65, no. 3 (2024): 420–432, 10.1007/s12016-023-08976-y. 38175322

[28] M. I. Gómez , A. Lee , B. Reddy , et al., “Staphylococcus Aureus Protein A Induces Airway Epithelial Inflammatory Responses by Activating TNFR1,” Nature Medicine 10, no. 8 (2004): 842–848, 10.1038/nm1079. 15247912

[29] M. Lötzerich , P. S. Roulin , K. Boucke , R. Witte , O. Georgiev , and U. F. Greber , “Rhinovirus 3C Protease Suppresses Apoptosis and Triggers Caspase‐Independent Cell Death,” Cell Death & Disease 9, no. 3 (2018): 272, 10.1038/s41419-018-0306-6. 29449668 PMC5833640

[30] M. H. Orzalli , A. Prochera , L. Payne , A. Smith , J. A. Garlick , and J. C. Kagan , “Virus‐Mediated Inactivation of Anti‐Apoptotic Bcl‐2 Family Members Promotes Gasdermin‐E‐Dependent Pyroptosis in Barrier Epithelial Cells,” Immunity 54, no. 7 (2021): 1447–1462.e5, 10.1016/j.immuni.2021.04.012. 33979579 PMC8594743

[31] J. Simpson , K. M. Spann , and S. Phipps , “MLKL Regulates Rapid Cell Death‐Independent HMGB1 Release in RSV Infected Airway Epithelial Cells,” Frontiers in Cell and Developmental Biology 10 (2022): 890389, 10.3389/fcell.2022.890389. 35712662 PMC9194532

[32] M. Li , Z. Fu , C. Qi , Q. Wang , H. Xie , and H. Li , “Some Macrophages With High Expression of CHOP Undergo Necroptosis in Chronic Rhinosinusitis,” American Journal of Rhinology & Allergy 37, no. 4 (2023): 449–455, 10.1177/19458924231163974. 36946083

[33] S. hang Wen , L. na Lin , H. jun Wu , et al., “TNF‐α Increases Staphylococcus Aureus‐Induced Death of Human Alveolar Epithelial Cell Line A549 Associated With RIP3‐Mediated Necroptosis,” Life Sciences 195 (2018): 81–86, 10.1016/j.lfs.2018.01.008. 29330116

[34] Y. J. Jeon , C. H. Gil , J. Won , A. Jo , and H. J. Kim , “Symbiotic Microbiome Staphylococcus Epidermidis Restricts IL‐33 Production in Allergic Nasal Epithelium via Limiting the Cellular Necroptosis,” BMC Microbiology 23, no. 1 (2023): 154, 10.1186/s12866-023-02898-7. 37237381 PMC10214541

[35] M. B. Wagner , J. Baker , R. G. Douglas , M. W. Taylor , and K. Biswas , “Detection and Quantification of *Staphylococcus* in Chronic Rhinosinusitis,” International Forum of Allergy & Rhinology 9, no. 12 (2019): 1462–1469, 10.1002/alr.22425. 31483577

[36] W. Zhang , Y. Lei , T. Zhang , et al., “IL‐8 Promotes Pyroptosis Through ERK Pathway and Mediates Glucocorticoid Resistance in Chronic Rhinosinusitis With Nasal Polyps,” Inflammation Research 74, no. 1 (2025): 20, 10.1007/s00011-024-01982-6. 39821406

[37] S. Shimizu , H. Kouzaki , T. Kato , I. Tojima , and T. Shimizu , “HMGB1‐TLR4 Signaling Contributes to the Secretion of Interleukin 6 and Interleukin 8 by Nasal Epithelial Cells,” American Journal of Rhinology & Allergy 30, no. 3 (2016): 167–172, 10.2500/ajra.2016.30.4300. 27216346

[38] B. Zhong , S. Sun , K. S. Tan , et al., “Hypoxia‐Inducible Factor 1α Activates the NLRP3 Inflammasome to Regulate Epithelial Differentiation in Chronic Rhinosinusitis,” Journal of Allergy and Clinical Immunology 152, no. 6 (2023): 1444–1459.e14, 10.1016/j.jaci.2023.09.020. 37777019

[39] J. Yao , Q. Kong , Y. Wang , Y. Zhang , and Q. Wang , “Mechanism of Kruppel‐Like Factor 4 in Pyroptosis of Nasal Mucosal Epithelial Cells in Mice With Allergic Rhinitis,” American Journal of Rhinology & Allergy 37, no. 3 (2023): 337–347, 10.1177/19458924221148568. 36799547

[40] D. Li , J. Zhang , L. Wang , et al., “Identification of Pyroptosis‐Related Genes Regulating the Progression of Chronic Rhinosinusitis With Nasal Polyps,” International Archives of Allergy and Immunology 185, no. 5 (2024): 411–424, 10.1159/000536371. 38402873

[41] T. Liu , Y. T. Zhou , L. Q. Wang , et al., “NOD‐Like Receptor Family, Pyrin Domain Containing 3 (NLRP3) Contributes to Inflammation, Pyroptosis, and Mucin Production in Human Airway Epithelium on Rhinovirus Infection,” Journal of Allergy and Clinical Immunology 144, no. 3 (2019): 777–787.e9, 10.1016/j.jaci.2019.05.006. 31102698

[42] Pei Y. Y. , Huang D. Y. , Zhang T. , et al., “Zhonghua Er Bi Yan Hou Tou Jing Wai Ke Za Zhi,” 2021;56(12):1328‐1335, 10.3760/cma.j.cn115330-20210125-00036. 34963222

[43] V. Brinkmann , B. Laube , U. Abu Abed , C. Goosmann , and A. Zychlinsky , “Neutrophil Extracellular Traps: How to Generate and Visualize Them,” Journal of Visualized Experiments, no. 36 (2010): 1724, 10.3791/1724-v. 20182410 PMC3125121

[44] N. Germic , D. Stojkov , K. Oberson , S. Yousefi , and H. Simon , “Neither Eosinophils Nor Neutrophils Require ATG 5‐Dependent Autophagy for Extracellular DNA Trap Formation,” Immunology 152, no. 3 (2017): 517–525, 10.1111/imm.12790. 28703297 PMC5629432

[45] Y. Cao , F. Chen , Y. Sun , et al., “LL‐37 Promotes Neutrophil Extracellular Trap Formation in Chronic Rhinosinusitis With Nasal Polyps,” Clinical and Experimental Allergy 49, no. 7 (2019): 990–999, 10.1111/cea.13408. 31046155

[46] H. Nagase , S. Ueki , and S. Fujieda , “The Roles of IL‐5 and anti‐IL‐5 Treatment in Eosinophilic Diseases: Asthma, Eosinophilic Granulomatosis With Polyangiitis, and Eosinophilic Chronic Rhinosinusitis,” Allergology International 69, no. 2 (2020): 178–186, 10.1016/j.alit.2020.02.002. 32139163

[47] J. Li , H. Zhao , J. Yang , et al., “The Role and Mechanism of Extracellular Traps in Chronic Rhinosinusitis,” Biomedicine & Pharmacotherapy 181 (2024): 117655, 10.1016/j.biopha.2024.117655. 39486368

[48] Z. Tu , M. Liu , C. Xu , et al., “Functional 2D Nanoplatforms Alleviate Eosinophilic Chronic Rhinosinusitis by Modulating Eosinophil Extracellular Trap Formation,” Advanced Science 11, no. 19 (2024): 2307800, 10.1002/advs.202307800. 38477549 PMC11109617

[49] A. J. Thomas , A. Pulsipher , B. M. Davis , and J. A. Alt , “LL‐37 Causes Cell Death of Human Nasal Epithelial Cells, Which Is Inhibited With a Synthetic Glycosaminoglycan,” in PLoS One, edited by N. A. Cohen , Vol. 12, (2017), 10.1371/journal.pone.0183542.8 e0183542 28837619 PMC5570287

[50] A. Alles , K. Alley , J. C. Barrett , et al., “Apoptosis: A General Comment,” FASEB Journal 5, no. 8 (1991): 2127–2128, 10.1096/fasebj.5.8.2022310. 2022310

[51] X. Xu , Y. Lai , and Z. C. Hua , “Apoptosis and Apoptotic Body: Disease Message and Therapeutic Target Potentials,” Bioscience Reports 39, no. 1 (2019): BSR20180992, 10.1042/bsr20180992. 30530866 PMC6340950

[52] T. Takahashi , A. Kato , S. Berdnikovs , et al., “Microparticles in Nasal Lavage Fluids in Chronic Rhinosinusitis: Potential Biomarkers for Diagnosis of Aspirin‐Exacerbated Respiratory Disease,” Journal of Allergy and Clinical Immunology 140, no. 3 (2017): 720–729, 10.1016/j.jaci.2017.01.022. 28238741 PMC5568994

[53] J. Cheng , J. Yang , K. Xue , et al., “Desmoglein 3 Silencing Inhibits Inflammation and Goblet Cell Mucin Secretion in a Mouse Model of Chronic Rhinosinusitis via Disruption of the Wnt/β‐Catenin Signaling Pathway,” Inflammation 42, no. 4 (2019): 1370–1382, 10.1007/s10753-019-00998-z. 31028575

[54] A. DіLcі , N. Varol , İ Kiliçcioğlu , et al., “Expression Profiles of CD11b, Galectin‐1, Beclin‐1, and Caspase‐3 in Nasal Polyposis,” Turkish Journal of Medical Sciences 47 (2017): 1757–1764, 10.3906/sag-1705-108. 29306235

[55] H. Hu , S. Wang , J. Wang , R. Huang , P. Dong , and Z. Sun , “uPA Affects the CRSsNP Nasal Mucosa Epithelium Apoptosis by Regulating WIF1,” Experimental Cell Research 377, no. 1–2 (2019): 75–85, 10.1016/j.yexcr.2018.12.024. 30605632

[56] M. Morawska‐Kochman , A. Śmieszek , K. Marcinkowska , et al., “Expression of Apoptosis‐Related Biomarkers in Inflamed Nasal Sinus Epithelium of Patients With Chronic Rhinosinusitis With Nasal Polyps (CRSwNP)—Evaluation at mRNA and miRNA Levels,” Biomedicines 10, no. 6 (2022): 1400, 10.3390/biomedicines10061400. 35740420 PMC9220377

[57] L. Meng , X. Qu , P. Tao , J. Dong , and R. Guo , “Quercetin Alleviates the Progression of Chronic Rhinosinusitis Without Nasal Polyps by Inhibiting Nasal Mucosal Inflammation and Epithelial Apoptosis,” Molecular Biotechnology (2024). 10.1007/s12033-024-01269-5 39240457

[58] Y. Verhoeven , S. Tilborghs , J. Jacobs , et al., “The Potential and Controversy of Targeting STAT Family Members in Cancer,” Seminars in Cancer Biology 60 (2020): 41–56, 10.1016/j.semcancer.2019.10.002. 31605750

[59] Linke R. , Pries R. , Könnecke M. , et al., “Increased Phosphorylation of STAT5b, But Not STAT5a,” American Journal of Rhinology & Allergy, Nasal Polyps 29, no. 3 (2020): 182–187, 10.2500/ajra.2015.29.4170. 25975249

[60] C. Liu , H. Du , Y. Wang , et al., “S100A11 Regulates Nasal Epithelial Cell Remodeling and Inflammation in CRSwNPs via the RAGE‐Mediated AMPK‐STAT3 Pathway,” Molecular Immunology 140 (2021): 35–46, 10.1016/j.molimm.2021.09.014. 34653793

[61] A. J. Levine , “p53, the Cellular Gatekeeper for Growth and Division,” Cell 88, no. 3 (1997): 323–331, 10.1016/s0092-8674(00)81871-1. 9039259

[62] M. Deng , C. Lin , X. Zeng , et al., “Involvement of p53, p21, and Caspase‐3 in Apoptosis of Coronary Artery Smooth Muscle Cells in a Kawasaki Vasculitis Mouse Model,” Medical Science Monitor 26 (2020), 10.12659/msm.922429. PMC7456161 32820144

[63] A. Lavezzi , M. Mantovani , A. Cazzullo , P. Turconi , and L. Matturri , p53 Over‐Expression and Its Correlation With PCNA Index in Nasal Polyps. 10670029

[64] D. S. Küpper , F. C. P. Valera , R. Malinsky , et al., “Expression of Apoptosis Mediators p53 and Caspase 3, 7, and 9 in Chronic Rhinosinusitis With Nasal Polyposis,” American Journal of Rhinology & Allergy 28, no. 3 (2014): 187–191, 10.2500/ajra.2014.28.4022. 24980229

[65] N. Yu Matveeva , D. G. Pavlush , and S. G. Kalinichenko , “Expression of Pro‐ and Anti‐Apoptotic Molecules in the Mucous Membrane of the Nasal Cavity With Polypous Rhinosinusitis,” Vestnik Otorinolaringologii 85, no. 3 (2020): 43, 10.17116/otorino20208503143. 32628382

[66] M. Dutsch‐Wicherek , R. Tomaszewska , P. Strek , L. Wicherek , and J. Skladzien , “[No title found],” BMC Immunology 7, no. 1 (2006): 4, 10.1186/1471-2172-7-4. 16551346 PMC1448183

[67] L. Haymour , M. Jean , C. Smulski , and P. Legembre , “CD95 (Fas) and CD95L (FasL)‐mediated Non‐Canonical Signaling Pathways,” Biochimica et Biophysica Acta (BBA)—Reviews on Cancer 1878, no. 6 (2023): 189004, 10.1016/j.bbcan.2023.189004. 37865305

[68] S. Mitamura , H. Ikawa , N. Mizuno , Y. Kaziro , and H. Itoh , “Cytosolic Nuclease Activated by Caspase‐3 and Inhibited by DFF‐45,” Biochemical and Biophysical Research Communications 243, no. 2 (1998): 480–484, 10.1006/bbrc.1998.8122. 9480834

[69] S. Y. Fang and B. C. Yang , “Overexpression of Fas‐Ligand in Human Nasal Polyps,” Annals of Otology, Rhinology & Laryngology 109, no. 3 (2000): 267–270, 10.1177/000348940010900306. 10737309

[70] M. L. C. Silveira , E. Tamashiro , A. R. D. Santos , et al., “miRNA‐205‐5p Can Be Related to T2‐polarity in Chronic Rhinosinusitis With Nasal Polyps,” Rhin (2021). 10.4193/Rhin21.109 34608897

[71] Z. Liu , H. Liu , D. Yu , J. Gao , B. Ruan , and R. Long , “Downregulation of miR‐29b‐3p Promotes α‐Tubulin Deacetylation by Targeting the Interaction of Matrix Metalloproteinase‐9 With Integrin β1 in Nasal Polyps,” International Journal of Molecular Medicine 48, no. 1 (2021): 126, 10.3892/ijmm.2021.4959. 33982786 PMC8128418

[72] J. Cheng , J. Chen , Y. Zhao , J. Yang , K. Xue , and Z. Wang , “MicroRNA‐761 Suppresses Remodeling of Nasal Mucosa and Epithelial–Mesenchymal Transition in Mice With Chronic Rhinosinusitis Through LCN2,” Stem Cell Research & Therapy 11, no. 1 (2020): 151, 10.1186/s13287-020-01598-7. 32272958 PMC7147028

[73] J. Chen , D. Liu , J. Yang , C. Jin , C. Zhao , and J. Cheng , “Epidermal Growth Factor Activates a Hypoxia‐Inducible Factor 1α–MicroRNA‐21 Axis to Inhibit Aquaporin 4 in Chronic Rhinosinusitis,” Annals of the New York Academy of Sciences 1518, no. 1 (2022): 299–314, 10.1111/nyas.14914. 36303271

[74] S. Volpe , J. Irish , S. Palumbo , et al., “Viral Infections and Chronic Rhinosinusitis,” Journal of Allergy and Clinical Immunology 152, no. 4 (2023): 819–826, 10.1016/j.jaci.2023.07.018. 37574080 PMC10592176

[75] D. Cantero , C. Cooksley , A. Bassiouni , et al., “ *Staphylococcus aureus* Biofilms Induce Apoptosis and Expression of Interferon‐γ, Interleukin‐10, and Interleukin‐17A on Human Sinonasal Explants,” American Journal of Rhinology & Allergy 29, no. 1 (2015): 23–28, 10.2500/ajra.2015.29.4130. 25590311

[76] H. Hu , S. Liu , K. Hon , A. J. Psaltis , P. J. Wormald , and S. Vreugde , “Staphylococcal Protein A Modulates Inflammation by Inducing Interferon Signaling in Human Nasal Epithelial Cells,” Inflammation Research 72, no. 2 (2023): 251–262, 10.1007/s00011-022-01656-1. 36527461 PMC9925485

[77] C. Ledo , C. D. Gonzalez , A. Garofalo , et al., “Protein A Modulates Neutrophil and Keratinocyte Signaling and Survival in Response to Staphylococcus aureus,” Frontiers in Immunology 11 (2021): 524180, 10.3389/fimmu.2020.524180. 33692774 PMC7937904

[78] Z. Yang , H. Mitländer , T. Vuorinen , and S. Finotto , “Mechanism of Rhinovirus Immunity and Asthma,” Frontiers in Immunology 12 (2021): 731846, 10.3389/fimmu.2021.731846. 34691038 PMC8526928

[79] Y. K. Ko , Y. L. Zhang , J. H. Wee , D. H. Han , H. J. Kim , and C. S. Rhee , “Human Rhinovirus Infection Enhances the Th2 Environment in Allergic and Non‐Allergic Patients With Chronic Rhinosinusitis,” Clinical and Experimental Otorhinolaryngology 14, no. 2 (2021): 217–224, 10.21053/ceo.2020.00444. 32911880 PMC8111390

[80] F. Zhu , Z. Teng , X. Zhou , et al., “H1N1 Influenza Virus‐Infected Nasal Mucosal Epithelial Progenitor Cells Promote Dendritic Cell Recruitment and Maturation,” Frontiers in Immunology 13 (2022): 879575, 10.3389/fimmu.2022.879575. 35572503 PMC9095954

[81] Z. Huang , J. Liu , L. Sun , et al., “Updated Epithelial Barrier Dysfunction in Chronic Rhinosinusitis: Targeting Pathophysiology and Treatment Response of Tight Junctions,” Allergy 79, no. 5 (2024): 1146–1165, 10.1111/all.16064. 38372149

[82] L. Vasilikos , L. M. Spilgies , J. Knop , and W. W. Wong , “Regulating the Balance Between Necroptosis, Apoptosis and Inflammation by Inhibitors of Apoptosis Proteins,” Immunology & Cell Biology 95, no. 2 (2017): 160–165, 10.1038/icb.2016.118. 27904150

[83] T. Tenev , M. Ditzel , A. Zachariou , and P. Meier , “The Antiapoptotic Activity of Insect IAPs Requires Activation by an Evolutionarily Conserved Mechanism,” Cell Death & Differentiation 14, no. 6 (2007): 1191–1201, 10.1038/sj.cdd.4402118. 17347664

[84] Z. F. Qiu , D. M. Han , L. Zhang , et al., “Expression of Survivin and Enhanced Polypogenesis in Nasal Polyps,” American Journal of Rhinology 22, no. 2 (2008): 106–110, 10.2500/ajr.2008.22.3139. 18336724

[85] N. Yun , C. Kim , H. Cha , et al., “Caspase‐3‐Mediated Cleavage of PICOT in Apoptosis,” Biochemical and Biophysical Research Communications 432, no. 3 (2013): 533–538, 10.1016/j.bbrc.2013.02.017. 23415866

[86] J. L. Allensworth , S. J. Sauer , H. K. Lyerly , M. A. Morse , and G. R. Devi , “Smac Mimetic Birinapant Induces Apoptosis and Enhances TRAIL Potency in Inflammatory Breast Cancer Cells in an IAP‐Dependent and TNF‐α‐Independent Mechanism,” Breast Cancer Research and Treatment 137, no. 2 (2013): 359–371, 10.1007/s10549-012-2352-6. 23225169

[87] T. Cakabay , I. Sayin , O. Erdur , A. Muhammedoglu , N. S. Tekke , and F. T. Kayhan , “Role of Apoptosis in the Pathogenesis of Nasal Polyps Based Upon Galectin‐3 Expression,” Journal of Craniofacial Surgery 28, no. 1 (2017): 280–284, 10.1097/scs.0000000000003174. 27922968

[88] Y. Zhao , N. Zhang , C. Perez Novo , Y. Wang , and L. Zhang , “Decreased Histone Expression in Chronic Rhinosinusitis With Nasal Polyps,” Asia Pacific Allergy, (2024). 10.5415/apallergy.0000000000000140 PMC11142755 38827263

[89] M. Dutsch‐Wicherek , “The Possible Biological Role of Metallothionein in Apoptosis,” Frontiers in Bioscience, no. 13 (2008): 4029, 10.2741/2991. 18508497

[90] M. Dutsch‐Wicherek , R. Tomaszewska , A. Lazar , et al., “The Evaluation of Metallothionein Expression in Nasal Polyps With Respect to Immune Cell Presence and Activity,” BMC Immunology 11, no. 1 (2010): 10, 10.1186/1471-2172-11-10. 20214821 PMC2848203

[91] H. S. Lee , A. Myers , and J. Kim , “Vascular Endothelial Growth Factor Drives Autocrine Epithelial Cell Proliferation and Survival in Chronic Rhinosinusitis With Nasal Polyposis,” American Journal of Respiratory and Critical Care Medicine 180, no. 11 (2009): 1056–1067, 10.1164/rccm.200905-0740oc. 19762561 PMC2784412

[92] F. Carsuzaa , É Béquignon , M. Bainaud , et al., “Oncostatin M Counteracts the Fibrotic Effects of TGF‐β1 and IL‐4 On Nasal‐Polyp‐Derived Fibroblasts: A Control of Fibrosis in Chronic Rhinosinusitis With Nasal Polyps?,” International Journal of Mathematics and Statistics 23, no. 11 (2022): 6308, 10.3390/ijms23116308. PMC9181333 35682987

[93] F. Wu , P. Tian , Y. Ma , J. Wang , H. Ou , and H. Zou , “Reactive Oxygen Species are Necessary for Bleomycin A5‐Induced Apoptosis and Extracellular Matrix Elimination of Nasal Polyp‐Derived Fibroblasts,” Annals of Otology, Rhinology & Laryngology 128, no. 2 (2019): 135–144, 10.1177/0003489418812905. 30450917

[94] S. Siddiqui , C. Bachert , L. Bjermer , et al., “Eosinophils and Tissue Remodeling: Relevance to Airway Disease,” Journal of Allergy and Clinical Immunology 152, no. 4 (2023): 841–857, 10.1016/j.jaci.2023.06.005. 37343842

[95] C. S. Lages , I. Lewkowich , A. Sproles , M. Wills‐Karp , and C. Chougnet , “Partial Restoration of T‐Cell Function in Aged Mice by In Vitro Blockade of the PD‐1/PD‐L1 Pathway,” Aging Cell 9, no. 5 (2010): 785–798, 10.1111/j.1474-9726.2010.00611.x. 20653631 PMC2941565

[96] Y. Han , D. Liu , and L. Li , PD‐1/PD‐L1 Pathway: Current Researches in Cancer. PMC7136921 32266087

[97] C. Bachert , A. Hicks , S. Gane , et al., “The Interleukin‐4/Interleukin‐13 Pathway in Type 2 Inflammation in Chronic Rhinosinusitis With Nasal Polyps,” Frontiers in Immunology 15 (2024): 1356298, 10.3389/fimmu.2024.1356298. 38690264 PMC11059040

[98] D. Qin , P. Liu , H. Zhou , et al., “TIM‐4 in Macrophages Contributes to Nasal Polyp Formation Through the TGF‐β1–mediated Epithelial to Mesenchymal Transition in Nasal Epithelial Cells,” Frontiers in Immunology 13 (2022): 941608, 10.3389/fimmu.2022.941608. 35990621 PMC9389014

[99] J. L. Larson‐Casey , J. S. Deshane , A. J. Ryan , V. J. Thannickal , and A. B. Carter , “Macrophage Akt1 Kinase‐Mediated Mitophagy Modulates Apoptosis Resistance and Pulmonary Fibrosis,” Immunity 44, no. 3 (2016): 582–596, 10.1016/j.immuni.2016.01.001. 26921108 PMC4794358

[100] C. Liu , K. Wang , W. Liu , J. Zhang , Y. Fan , and Y. Sun , “ALOX15+ M2 Macrophages Contribute to Epithelial Remodeling in Eosinophilic Chronic Rhinosinusitis With Nasal Polyps,” Journal of Allergy and Clinical Immunology 154, no. 3 (2024): 592–608, 10.1016/j.jaci.2024.04.019. 38705258

[101] R. Khalmuratova , M. Lee , J. H. Mo , Y. Jung , J. W. Park , and H. W. Shin , “Wogonin Attenuates Nasal Polyp Formation by Inducing Eosinophil Apoptosis Through HIF‐1α and Survivin Suppression,” Scientific Reports 8, no. 1 (2018): 6201, 10.1038/s41598-018-24356-5. 29670184 PMC5906673

[102] B. A. Youngblood , J. Leung , R. Falahati , et al., “Discovery, Function, and Therapeutic Targeting of Siglec‐8,” Cells 10, no. 1 (2020): 19, 10.3390/cells10010019. 33374255 PMC7823959

[103] K. M. Buchheit , E. Lewis , D. Gakpo , et al., “Mepolizumab Targets Multiple Immune Cells in Aspirin‐Exacerbated Respiratory Disease,” Journal of Allergy and Clinical Immunology 148, no. 2 (2021): 574–584, 10.1016/j.jaci.2021.05.043. 34144111 PMC9096876

[104] P. Gevaert , J. K. Han , S. G. Smith , et al., “The Roles of Eosinophils and Interleukin‐5 in the Pathophysiology of Chronic Rhinosinusitis With Nasal Polyps,” International Forum of Allergy & Rhinology 12, no. 11 (2022): 1413–1423, 10.1002/alr.22994. 35243803 PMC9790271

[105] M. L. Kowalski , J. Grzegorczyk , R. Pawliczak , T. Kornatowski , M. Wagrowska‐Danilewicz , and M. Danilewicz , “Decreased Apoptosis and Distinct Profile of Infiltrating Cells in the Nasal Polyps of Patients With Aspirin Hypersensitivity,” Allergy 57, no. 6 (2002): 493–500, 10.1034/j.1398-9995.2002.13508.x. 12028114

[106] T. Kiwamoto , N. Kawasaki , J. C. Paulson , and B. S. Bochner , “Siglec‐8 as a Drugable Target to Treat Eosinophil and Mast Cell‐Associated Conditions,” Pharmacology & Therapeutics 135, no. 3 (2012): 327–336, 10.1016/j.pharmthera.2012.06.005. 22749793 PMC3587973

[107] D. K. Kim , H. S. Lim , K. M. Eun , et al., “Subepithelial Neutrophil Infiltration as a Predictor of the Surgical Outcome of Chronic Rhinosinusitis With Nasal Polyps,” Rhin (2020). 10.4193/Rhin20.373 33129200

[108] J. W. Ruan , J. F. Zhao , X. L. Li , et al., “Corrigendum: Characterizing the Neutrophilic Inflammation in Chronic Rhinosinusitis With Nasal Polyps,” Frontiers in Cell and Developmental Biology 12 (2024): 1450040, 10.3389/fcell.2024.1450040. 39092187 PMC11293497

[109] S. Van Nevel , J. Declercq , G. Holtappels , B. N. Lambrecht , and C. Bachert , “Granulocyte‐Colony Stimulating Factor: Missing Link for Stratification of Type 2–High and Type 2–low Chronic Rhinosinusitis Patients,” Journal of Allergy and Clinical Immunology 149, no. 5 (2022): 1655–1665.e5, 10.1016/j.jaci.2022.02.019. 35278495

[110] J. W. Ruan , J. F. Zhao , X. L. Li , et al., “Characterizing the Neutrophilic Inflammation in Chronic Rhinosinusitis With Nasal Polyps,” Frontiers in Cell and Developmental Biology 9 (2021): 793073, 10.3389/fcell.2021.793073. 34977034 PMC8718617

[111] A. Kimura and T. Kishimoto , “IL‐6: Regulator of Treg/Th17 Balance,” European Journal of Immunology 40, no. 7 (2010): 1830–1835, 10.1002/eji.201040391. 20583029

[112] M. C. Scavuzzo , B. Fattori , R. Ruffoli , et al., “Inflammatory Mediators and Eosinophilia in Atopic and Non‐Atopic Patients With Nasal Polyposis,” Biomedicine & Pharmacotherapy 59, no. 6 (2005): 323–329, 10.1016/j.biopha.2004.11.010. 15935609

[113] R. Linke , R. Pries , M. Könnecke , et al., “Glycogen Synthase Kinase 3 in Chronic Rhinosinusitis: Two Faces of a Single Enzyme in One Disease,” Annals of Allergy, Asthma, & Immunology 110, no. 2 (2013): 101–106, 10.1016/j.anai.2012.11.016. 23352529

[114] O. G. Ahmed and N. R. Rowan , “Olfactory Dysfunction and Chronic Rhinosinusitis,” Immunology and Allergy Clinics of North America 40, no. 2 (2020): 223–232, 10.1016/j.iac.2019.12.013. 32278447

[115] W. H. Huang , Y. W. Hung , W. Hung , M. Y. Lan , and C. F. Yeh , “Murine Model of Eosinophilic Chronic Rhinosinusitis With Nasal Polyposis Inducing Neuroinflammation and Olfactory Dysfunction,” Journal of Allergy and Clinical Immunology 154, no. 2 (2024): 325–339.e3, 10.1016/j.jaci.2024.02.021. 38494093

[116] S. Becker , C. Pflugbeil , M. Gröger , M. Canis , G. J. Ledderose , and M. F. Kramer , “Olfactory Dysfunction in Seasonal and Perennial Allergic Rhinitis,” Acta Oto‐Laryngologica 132, no. 7 (2012): 763–768, 10.3109/00016489.2012.656764. 22497546

[117] Y. Suzuki and A. I. Farbman , “Tumor Necrosis Factor‐α‐Induced Apoptosis in Olfactory Epithelium In Vitro: Possible Roles of Caspase 1 (ICE), Caspase 2 (ICH‐1), and Caspase 3 (CPP32),” Experimental Neurology 165, no. 1 (2000): 35–45, 10.1006/exnr.2000.7465. 10964483

[118] T. Pozharskaya , J. Liang , and A. P. Lane , “Regulation of Inflammation‐Associated Olfactory Neuronal Death and Regeneration by the Type II Tumor Necrosis Factor Receptor,” International Forum of Allergy & Rhinology 3, no. 9 (2013): 740–747, 10.1002/alr.21187. 23733314 PMC3784625

[119] B. Sultan , L. A. May , and A. P. Lane , “The Role of Tnf‐Α in Inflammatory Olfactory Loss,” Laryngoscope 121, no. 11 (2011): 2481–2486, 10.1002/lary.22190. 21882204 PMC3540407

[120] N. M. Gangadhar , S. J. Firestein , and B. R. Stockwell , “A Novel Role for Jun N‐terminal Kinase Signaling in Olfactory Sensory Neuronal Death,” Molecular and Cellular Neuroscience 38, no. 4 (2008): 518–525, 10.1016/j.mcn.2008.04.013. 18571430 PMC2568995

[121] S. Papa , F. Zazzeroni , C. G. Pham , C. Bubici , and G. Franzoso , “Linking JNK Signaling to NF‐κB: A Key to Survival,” Journal of Cell Science 117, no. 22 (2004): 5197–5208, 10.1242/jcs.01483. 15483317

[122] H. Lv , P. Liu , F. Zhou , Z. Gao , W. Fan , and Y. Xu , “TAK‐242 Ameliorates Olfactory Dysfunction in a Mouse Model of Allergic Rhinitis by Inhibiting Neuroinflammation in the Olfactory Bulb,” International Immunopharmacology 92 (2021): 107368, 10.1016/j.intimp.2021.107368. 33454639

[123] H. Hoblos , W. Cawthorne , A. L. Samson , and J. M. Murphy , “Protein Shapeshifting in Necroptotic Cell Death Signaling,” Trends in Biochemical Sciences 50, no. 2 (2025): 92–105, 10.1016/j.tibs.2024.11.006. 39730228

[124] S. P. Goldie , L. C. Lau , H. A. S. Jones , P. G. Harries , A. F. Walls , and R. J. Salib , “Identification of Novel Staphylococcus aureus Core and Accessory Virulence Patterns in Chronic Rhinosinusitis,” International Journal of Molecular Sciences 26, no. 8 (2025): 3711, 10.3390/ijms26083711. 40332362 PMC12027640

[125] Kitur K. , Parker D. , Nieto P. , et al., “Toxin‐Induced Necroptosis is a Major Mechanism of Staphylococcus aureus Lung Damage.” in PLoS Pathogens, edited by Miller L. S. 11(4) (2015):e1004820, 10.1371/journal.ppat.1004820. 25880560 PMC4399879

[126] Y. Yao , Y. Wang , Z. Zhang , et al., “Chop Deficiency Protects Mice Against Bleomycin‐induced Pulmonary Fibrosis by Attenuating M2 Macrophage Production,” Molecular Therapy 24, no. 5 (2016): 915–925, 10.1038/mt.2016.36. 26883801 PMC4881771

[127] Y. Wang , J. Zhu , L. Zhang , et al., “Role of C/EBP Homologous Protein and Endoplasmic Reticulum Stress in Asthma Exacerbation by Regulating the IL‐4/signal Transducer and Activator of Transcription 6/Transcription Factor EC/IL‐4 Receptor α Positive Feedback Loop in M2 Macrophages,” Journal of Allergy and Clinical Immunology 140, no. 6 (2017): 1550–1561.e8, 10.1016/j.jaci.2017.01.024. 28238747

[128] E. P. S. Lam , H. H. Kariyawasam , B. M. J. Rana , et al., “IL‐25/IL‐33‐Responsive TH2 Cells Characterize Nasal Polyps With a Default TH17 Signature in Nasal Mucosa,” Journal of Allergy and Clinical Immunology 137, no. 5 (2016): 1514–1524, 10.1016/j.jaci.2015.10.019. 26684290 PMC4852988

[129] N. Oikonomou , M. J. Schuijs , A. Chatzigiagkos , et al., “Airway Epithelial Cell Necroptosis Contributes to Asthma Exacerbation in a Mouse Model of House Dust Mite‐Induced Allergic Inflammation,” Mucosal Immunology 14, no. 5 (2021): 1160–1171, 10.1038/s41385-021-00415-5. 34045680 PMC8379077

[130] S. H. Yeon , G. Yang , H. E. Lee , and J. Y. Lee , “Oxidized Phosphatidylcholine Induces the Activation of NLRP3 Inflammasome in Macrophages,” Journal of Leukocyte Biology 101, no. 1 (2017): 205–215, 10.1189/jlb.3vma1215-579rr. 27256568

[131] A. A. Bhat , R. Thapa , O. Afzal , et al., “The Pyroptotic Role of Caspase‐3/GSDME Signalling Pathway Among Various Cancer: A Review,” International Journal of Biological Macromolecules 242 (2023): 124832, 10.1016/j.ijbiomac.2023.124832. 37196719

[132] H. Zhou , L. Wang , W. Lv , and H. Yu , “The NLRP3 Inflammasome in Allergic Diseases: Mechanisms and Therapeutic Implications,” Clinical and Experimental Medicine 24, no. 1 (2024): 231, 10.1007/s10238-024-01492-z. 39325206 PMC11427518

[133] M. Ding , X. Wei , C. Liu , and X. Tan , “Mahuang Fuzi Xixin Decoction Alleviates Allergic Rhinitis by Inhibiting NLRP3/Caspase‐1/GSDMD‐N‐Mediated Pyroptosis,” Journal of Ethnopharmacology 327 (2024): 118041, 10.1016/j.jep.2024.118041. 38479543

[134] J. Xu , J. Li , X. Wang , et al., “IRF4 Knockdown Inhibits the Chronic Rhinosinusitis Without Nasal Polyps Development by Regulating NLRP3/Caspase‐1/GSDMD‐Mediated Pyroptosis,” Biochemical Genetics 63, no. 2 (2025): 1880–1900, 10.1007/s10528-024-10792-8. 38635014

[135] Q. Wang , W. Wen , L. Zhou , et al., “LL‐37 Improves Sepsis‐Induced Acute Lung Injury by Suppressing Pyroptosis in Alveolar Epithelial Cells,” International Immunopharmacology 129 (2024): 111580, 10.1016/j.intimp.2024.111580. 38310763

[136] L. Chang , H. Wu , W. Huang , et al., “IL‐21 Induces Pyroptosis of Treg Cells via Akt–mTOR–NLRP3–Caspase 1 Axis in Eosinophilic Chronic Rhinosinusitis,” Journal of Allergy and Clinical Immunology 152, no. 3 (2023): 641–655.e14, 10.1016/j.jaci.2023.04.013. 37164271

[137] D. Simon , H.‐U. Simon , and S. Yousefi , “Extracellular DNA Traps in Allergic, Infectious, and Autoimmune Diseases,” Allergy 68, no. 4 (2013): 409–416, 10.1111/all.12111. 23409745

[138] S. Lim , R. Khalmuratova , Y. Y. Lee , et al., “Neutrophil Extracellular Traps Promote ΔNp63+ Basal Cell Hyperplasia in Chronic Rhinosinusitis,” Journal of Allergy and Clinical Immunology 153, no. 3 (2024): 705–717.e11, 10.1016/j.jaci.2023.11.016. 38000697

[139] H. Cha , H. S. Lim , J. A. Park , et al., “Effects of Neutrophil and Eosinophil Extracellular Trap Formation on Refractoriness in Chronic Rhinosinusitis With Nasal Polyps,” Allergy, Asthma & Immunology Research 15, no. 1 (2023): 94, 10.4168/aair.2023.15.1.94. PMC9880302 36693361

[140] C. S. Hwang , S. C. Park , H. J. Cho , D. J. Park , J. H. Yoon , and C. H. Kim , “Eosinophil Extracellular Trap Formation Is Closely Associated With Disease Severity in Chronic Rhinosinusitis Regardless of Nasal Polyp Status,” Scientific Reports 9, no. 1 (2019): 8061, 10.1038/s41598-019-44627-z. 31147604 PMC6542829

[141] T. Delemarre , G. Holtappels , N. De Ruyck , et al., “A Substantial Neutrophilic Inflammation as Regular Part of Severe Type 2 Chronic Rhinosinusitis With Nasal Polyps,” Journal of Allergy and Clinical Immunology 147, no. 1 (2021): 179–188.e2, 10.1016/j.jaci.2020.08.036. 32949587

[142] S. Ueki , T. Tokunaga , S. Fujieda , et al., “Eosinophil Etosis and DNA Traps: A New Look at Eosinophilic Inflammation,” Current Allergy and Asthma Reports 16, no. 8 (2016): 54, 10.1007/s11882-016-0634-5. 27393701 PMC5313036

[143] E. Gevaert , N. Zhang , O. Krysko , et al., “Extracellular Eosinophilic Traps in Association With Staphylococcus aureus at the Site of Epithelial Barrier Defects in Patients With Severe Airway Inflammation,” Journal of Allergy and Clinical Immunology 139, no. 6 (2017): 1849–1860.e6, 10.1016/j.jaci.2017.01.019. 28216437

[144] M. Fritsch , S. D. Günther , R. Schwarzer , et al., “Caspase‐8 Is the Molecular Switch for Apoptosis, Necroptosis and Pyroptosis,” Nature 575, no. 7784 (2019): 683–687, 10.1038/s41586-019-1770-6. 31748744

[145] R. Wang , Y. Wang , H. Liu , et al., “Platycodon D Protects Human Nasal Epithelial Cells From Pyroptosis Through the Nrf2/HO‐1/ROS Signaling Cascade in Chronic Rhinosinusitis,” Chinese Medicine 19, no. 1 (2024): 40, 10.1186/s13020-024-00897-y. 38433216 PMC10910709

[146] B. Sundaram and T. D. Kanneganti , “Advances in Understanding Activation and Function of the NLRC4 Inflammasome,” International Journal of Mathematics and Statistics 22, no. 3 (2021): 1048, 10.3390/ijms22031048. PMC7864484 33494299

[147] Y. Cao , M. Shi , L. Liu , et al., “Inhibition of Neutrophil Extracellular Trap Formation Attenuates NLRP1‐Dependent Neuronal Pyroptosis via STING/IRE1α Pathway After Traumatic Brain Injury in Mice,” Frontiers in Immunology 14 (2023): 1125759, 10.3389/fimmu.2023.1125759. 37143681 PMC10152368

[148] K. W. Chen , M. Monteleone , D. Boucher , et al., “Noncanonical Inflammasome Signaling Elicits Gasdermin D–Dependent Neutrophil Extracellular Traps,” Science Immunology 3, no. 26 (2018): eaar6676, 10.1126/sciimmunol.aar6676. 30143554

[149] J. S. Silveira , G. L. Antunes , D. B. Kaiber , et al., “Autophagy Induces Eosinophil Extracellular Traps Formation and Allergic Airway Inflammation in a Murine Asthma Model,” Journal of Cellular Physiology 235, no. 1 (2020): 267–280, 10.1002/jcp.28966. 31206674

[150] J. Desai , O. Foresto‐Neto , M. Honarpisheh , et al., “Particles of Different Sizes and Shapes Induce Neutrophil Necroptosis Followed by the Release of Neutrophil Extracellular Trap‐Like Chromatin,” Scientific Reports 7, no. 1 (2017): 15003, 10.1038/s41598-017-15106-0. 29101355 PMC5670218

[151] N. Pandian and T. D. Kanneganti , “Panoptosis: A Unique Innate Immune Inflammatory Cell Death Modality,” Journal of Immunology 209, no. 9 (2022): 1625–1633, 10.4049/jimmunol.2200508. PMC9586465 36253067

[152] P. Samir , R. K. S. Malireddi , and T. D. Kanneganti , “The PANoptosome: A Deadly Protein Complex Driving Pyroptosis, Apoptosis, and Necroptosis (PANoptosis),” Frontiers in Cellular and Infection Microbiology 10 (2020): 238, 10.3389/fcimb.2020.00238. 32582562 PMC7283380

[153] X. Sun , Y. Yang , X. Meng , J. Li , X. Liu , and H. Liu , “PANoptosis: Mechanisms, Biology, and Role in Disease,” Immunological Reviews 321, no. 1 (2024): 246–262, 10.1111/imr.13279. 37823450

[154] R. Karki , B. R. Sharma , S. Tuladhar , et al., “Synergism of TNF‐α and IFN‐γ Triggers Inflammatory Cell Death, Tissue Damage, and Mortality in SARS‐CoV‐2 Infection and Cytokine Shock Syndromes,” Cell 184, no. 1 (2021): 149–168.e17, 10.1016/j.cell.2020.11.025. 33278357 PMC7674074

[155] S. Chen , J. Jiang , T. Li , and L. Huang , “PANoptosis: Mechanism and Role in Pulmonary Diseases,” International Journal of Molecular Sciences 24, no. 20 (2023): 15343, 10.3390/ijms242015343. 37895022 PMC10607352

[156] B. Yang , A. Hu , T. Wang , et al., “SARS‐CoV‐2 Infection Induces ZBP1‐Dependent PANoptosis in Bystander Cells,” Proceedings of the National Academy of Sciences of the United States of America 122, no. 28 (2025): e2500208122, 10.1073/pnas.2500208122. 40627395 PMC12280982

[157] R. E. Tweedell , T. Hibler , and T. D. Kanneganti , “Defining PANoptosis: Biochemical and Mechanistic Evaluation of Innate Immune Cell Death Activation,” Current Protocols 4, no. 7 (2024): e1112, 10.1002/cpz1.1112. 39073015 PMC11581195

[158] J. Wang , Y. Zhao , J. Ren , et al., “Heat Shock Protein 70 Is Induced by Pepsin via MAPK Signaling in Human Nasal Epithelial Cells,” European Archives of Oto‐Rhino‐Laryngology 276, no. 3 (2019): 767–774, 10.1007/s00405-018-5254-3. 30600344

[159] I. B. Németh , O. Zsíros , A. Koreck , et al., “Ultraviolet Light and Photodynamic Therapy Induce Apoptosis in Nasal Polyps,” Journal of Photochemistry and Photobiology B: Biology 117 (2012): 179–184, 10.1016/j.jphotobiol.2012.09.012. 23142931

[160] H. W. Cho , S. K. Park , K. W. Heo , and D. Y. Hur , “Methotrexate Induces Apoptosis in Nasal Polyps *via* Caspase Cascades and Both Mitochondria‐Mediated and p38 Mitogen‐Activated Protein Kinases/Jun N‐Terminal Kinase Pathways,” American Journal of Rhinology & Allergy 27, no. 1 (2013): e26–e31, 10.2500/ajra.2013.27.3849. 23406595

[161] X. Gao , N. Li , and J. Zhang , “SB203580, a p38MAPK Inhibitor, Attenuates Olfactory Dysfunction by Inhibiting OSN Apoptosis in AR Mice (Activation and Involvement of the p38 Mitogen‐Activated Protein Kinase in Olfactory Sensory Neuronal Apoptosis of Ova‐Induced Allergic Rhinitis),” Brain and Behavior 9, no. 6 (2019): e01295, 10.1002/brb3.1295. 31041850 PMC6577615

[162] S. Bobic , C. M. Van Drunen , I. Callebaut , et al., “Dexamethasone‐Induced Apoptosis of Freshly Isolated Human Nasal Epithelial Cells Concomitant With Abrogation of IL‐8 Production,” Rhin 48, no. 4 (2010): 401–407, 10.4193/rhino10.033. 21442075

[163] N. Žigart and Z. Časar , “A Literature Review of the Patent Publications on Venetoclax – A Selective Bcl‐2 Inhibitor: Discovering the Therapeutic Potential of a Novel Chemotherapeutic Agent,” Expert Opinion on Therapeutic Patents 29, no. 7 (2019): 487–496, 10.1080/13543776.2019.1627327. 31154862

[164] X. Geng , X. Wang , M. Luo , et al., “Induction of Neutrophil Apoptosis by a Bcl‐2 Inhibitor Reduces Particulate Matter‐Induced Lung Inflammation,” Aging 10, no. 6 (2018): 1415–1423, 10.18632/aging.101477. 29944468 PMC6046239

[165] B. P. Tian , F. Li , R. Li , et al., “Nanoformulated ABT‐199 to Effectively Target Bcl‐2 at Mitochondrial Membrane Alleviates Airway Inflammation by Inducing Apoptosis,” Biomaterials 192 (2019): 429–439, 10.1016/j.biomaterials.2018.06.020. 30500724 PMC6561093

[166] F. Wu , P. Tian , Y. Ma , J. Wang , H. Ou , and H. Zou , “Induction of Apoptosis in Nasal Polyp‐Derived Fibroblasts by Bleomycin A5 Inï¿½Vitro,” Molecular Medicine Reports (2018). 10.3892/mmr.2018.8540 29393498

[167] F. Wu , Y. Ma , J. Wang , et al., “Bleomycin A5 Suppresses Drp1‐Mediated Mitochondrial Fission and Induces Apoptosis in Human Nasal Polyp‐Derived Fibroblasts,” International Journal of Molecular Medicine 47, no. 1 (2020): 346–360, 10.3892/ijmm.2020.4797. 33236140 PMC7723402

[168] J. Shi , L. Dai , J. Gu , et al., “Luteolin Alleviates Olfactory Dysfunction in Eosinophilic Chronic Rhinosinusitis Through Modulation of the TLR4/NF‐κB Signaling Pathway,” International Immunopharmacology 148 (2025): 114189, 10.1016/j.intimp.2025.114189. 39892170

[169] A. Iwata , K. Nishio , R. K. Winn , E. Y. Chi , W. R. Henderson , and J. M. Harlan , “A Broad‐Spectrum Caspase Inhibitor Attenuates Allergic Airway Inflammation in Murine Asthma Model,” Journal of Immunology 170, no. 6 (2003): 3386–3391, 10.4049/jimmunol.170.6.3386. 12626599

[170] S. C. Sozmen , M. Karaman , S. C. Micili , et al., “Effects of Quercetin Treatment on Epithelium‐Derived Cytokines and Epithelial Cell Apoptosis in Allergic Airway Inflammation Micec,” Model 15, no. 6 (2016), PMID: 28129682. 28129681

[171] J. A. O’Sullivan , D. J. Carroll , Y. Cao , A. N. Salicru , and B. S. Bochner , “Leveraging Siglec‐8 Endocytic Mechanisms to Kill Human Eosinophils and Malignant Mast Cells,” Journal of Allergy and Clinical Immunology 141, no. 5 (2018): 1774–1785.e7, 10.1016/j.jaci.2017.06.028. 28734845 PMC6445644

[172] H. H. Walford , S. J. Lund , R. E. Baum , et al., “Increased ILC2s in the Eosinophilic Nasal Polyp Endotype Are Associated With Corticosteroid Responsiveness,” Clinical Immunology 155, no. 1 (2014): 126–135, 10.1016/j.clim.2014.09.007. 25236785 PMC4254351

[173] M. Takanosawa , H. Nishino , Y. Ohta , and K. Ichimura , “Glucocorticoids Enhance Regeneration of Murine Olfactory Epithelium,” Acta Oto‐Laryngologica 129, no. 9 (2009): 1002–1009, 10.1080/00016480802530663. 19016360

[174] H. Li , Y. Guan , B. Liang , et al., “Therapeutic Potential of MCC950, a Specific Inhibitor of NLRP3 Inflammasome,” European Journal of Pharmacology 928 (2022): 175091, 10.1016/j.ejphar.2022.175091. 35714692

[175] W. Zhang , G. Ba , R. Tang , M. Li , and H. Lin , “Ameliorative Effect of Selective NLRP3 Inflammasome Inhibitor MCC950 in An Ovalbumin‐Induced Allergic Rhinitis Murine Model,” International Immunopharmacology 83 (2020): 106394, 10.1016/j.intimp.2020.106394. 32193102

[176] J. Wang , C. Ren , W. Bi , and W. Batu , “Glycyrrhizin Mitigates Acute Lung Injury by Inhibiting the NLRP3 Inflammasome in Vitro and in Vivo,” Journal of Ethnopharmacology 303 (2023): 115948, 10.1016/j.jep.2022.115948. 36423713

[177] R. Wang , Y. Wang , Q. Yang , et al., “Xiaoqinglong Decoction Improves Allergic Rhinitis by Inhibiting NLRP3‐Mediated Pyroptosis in BALB/C Mice,” Journal of Ethnopharmacology 321 (2024): 117490, 10.1016/j.jep.2023.117490. 38030025

[178] W. Qin , X. Wu , Y. Jia , et al., “Suhuang Antitussive Capsule Inhibits NLRP3 Inflammasome Activation and Ameliorates Pulmonary Dysfunction via Suppression of Endoplasmic Reticulum Stress in Cough Variant Asthma,” Biomedicine & Pharmacotherapy 118 (2019): 109188, 10.1016/j.biopha.2019.109188. 31315072

[179] J. Lv , Y. Zhou , J. Wang , et al., “Heme oxygenase‐1 Alleviates Allergic Airway Inflammation by Suppressing NF‐κB ‐Mediated Pyroptosis of Bronchial Epithelial Cells,” FASEB Journal 38, no. 3 (2024): e23472, 10.1096/fj.202300883rr. 38329323

[180] J. Lv , M. Wu , and Z. Xia , “Heme Oxygenase‐1 Binds Gasdermin D to Inhibit Airway Epithelium Pyroptosis in Allergic Asthma,” Apoptosis 29, no. 11–12 (2024): 1853–1855, 10.1007/s10495-024-02016-6. 39190204

[181] L. Liu , L. Zhou , L. Wang , et al., “MUC1 Attenuates Neutrophilic Airway Inflammation in Asthma by Reducing NLRP3 Inflammasome‐Mediated Pyroptosis Through the Inhibition of the TLR4/MyD88/NF‐κB Pathway,” Respiratory Research 24, no. 1 (2023): 255, 10.1186/s12931-023-02550-y. 37880668 PMC10601133

[182] J. Yang , M. Zhang , Y. Luo , et al., “Protopine Ameliorates OVA‐Induced Asthma Through ModulatingTLR4/MyD88/NF‐κB Pathway and NLRP3 Inflammasome‐Mediated Pyroptosis,” Phytomedicine 126 (2024): 155410, 10.1016/j.phymed.2024.155410. 38367422

[183] S. H. Lee , M. R. Choi , J. Chung , S. H. Choi , S. K. Park , and Y. M. Kim , “Povidone Iodine Suppresses LPS‐Induced Inflammation by Inhibiting TLR4/MyD88 Formation in Airway Epithelial Cells,” Scientific Reports 12, no. 1 (2022): 3681, 10.1038/s41598-022-07803-2. 35256715 PMC8901750

[184] L. Cavone , C. Cuppari , S. Manti , et al., “Increase in the Level of Proinflammatory Cytokine HMGB1 in Nasal Fluids of Patients With Rhinitis and Its Sequestration by Glycyrrhizin Induces Eosinophil Cell Death,” Clin Exp Otorhinolaryngol 8, no. 2 (2015): 123, 10.3342/ceo.2015.8.2.123. 26045910 PMC4451536

[185] M. Zhang , L. Lian , T. Wang , et al., “Experimental and Proteomics Evidence Revealed the Protective Mechanisms of Shemazhichuan Liquid in Attenuating Neutrophilic Asthma,” Phytomedicine 135 (2024): 156180, 10.1016/j.phymed.2024.156180. 39515107

[186] P. Vandenabeele , S. Grootjans , N. Callewaert , and N. Takahashi , “Necrostatin‐1 Blocks Both RIPK1 and IDO: Consequences for the Study of Cell Death in Experimental Disease Models,” Cell Death & Differentiation 20, no. 2 (2013): 185–187, 10.1038/cdd.2012.151. 23197293 PMC3554339

[187] H. Zhang , Q. Liu , L. Kong , and S. Xu , “Mucin 1 Downregulation Impairs the Anti‐Necroptotic Effects of Glucocorticoids in Human Bronchial Epithelial Cells,” Life Sciences 221 (2019): 168–177, 10.1016/j.lfs.2019.02.013. 30738043

[188] X. A. Han , H. Y. Jie , J. H. Wang , et al., “Necrostatin‐1 Ameliorates Neutrophilic Inflammation in Asthma by Suppressing MLKL Phosphorylation to Inhibiting NETs Release,” Frontiers in Immunology 11 (2020): 666, 10.3389/fimmu.2020.00666. 32391007 PMC7194114

[189] S. G. Kelsen , I. O. Agache , W. Soong , et al., “Astegolimab (anti‐ST2) Efficacy and Safety in Adults With Severe Asthma: A Randomized Clinical Trial,” Journal of Allergy and Clinical Immunology 148, no. 3 (2021): 790–798, 10.1016/j.jaci.2021.03.044. 33872652

[190] D. J. Slade , S. Horibata , S. A. Coonrod , and P. R. Thompson , “A Novel Role for Protein Arginine Deiminase 4 in Pluripotency: The Emerging Role of Citrullinated Histone H1 in Cellular Programming,” BioEssays 36, no. 8 (2014): 736–740, 10.1002/bies.201400057. 24889365 PMC4151298

[191] Y. Chen , X. Liu , Y. Li , C. Quan , L. Zheng , and K. Huang , “Lung Cancer Therapy Targeting Histone Methylation: Opportunities and Challenges,” Computational and Structural Biotechnology Journal 16 (2018): 211–223, 10.1016/j.csbj.2018.06.001. 30002791 PMC6039709

[192] B. Peng , M. Y. Yan , Y. R. Chen , F. Sun , X. D. Xiang , and D. Liu , “The Methyl‐CpG Binding Domain 2 Regulates Peptidylarginine Deiminase 4 Expression and Promotes Neutrophil Extracellular Trap Formation via the Janus Kinase 2 Signaling Pathway in Experimental Severe Asthma,” Annals of Medicine 57, no. 1 (2025): 2458207, 10.1080/07853890.2025.2458207. 39865866 PMC11774153

[193] Chen Y. R. , Xiang X. D. , Sun F. , et al., “Simvastatin Reduces NETosis to Attenuate Severe Asthma by Inhibiting PAD4 Expression,” in Oxidative Medicine and Cellular Longevity edited by, Węgrzyn G. 2023 (2023): 1–13, 10.1155/2023/1493684. PMC9911252 36778209

[194] E. Zwiers , D. Montizaan , A. Kip , et al., “Inhibition of EETosis With an Anti‐Citrullinated Histone Antibody: A Novel Therapeutic Approach for Eosinophilic Inflammatory Disorders,” Frontiers in Immunology 16 (2025): 1533407, 10.3389/fimmu.2025.1533407. 40051617 PMC11882434

[195] B. Curren , T. Ahmed , D. R. Howard , et al., “IL‐33‐induced Neutrophilic Inflammation and NETosis Underlie Rhinovirus‐Triggered Exacerbations of Asthma,” Mucosal Immunology 16, no. 5 (2023): 671–684, 10.1016/j.mucimm.2023.07.002. 37506849

[196] M. Cazzola , D. Stolz , P. Rogliani , and M. G. Matera , “α _1_ ‐Antitrypsin Deficiency and Chronic Respiratory Disorders,” European Respiratory Review 29, no. 155 (2020): 190073, 10.1183/16000617.0073-2019. 32051168 PMC9488707

[197] X. Bai , J. Hippensteel , A. Leavitt , et al., “Hypothesis: Alpha‐1‐Antitrypsin Is a Promising Treatment Option for COVID‐19,” Medical Hypotheses 146 (2021): 110394, 10.1016/j.mehy.2020.110394. 33239231 PMC7659642

[198] T. J. Craig and M. P. Henao , “Advances in Managing COPD Related to α _1_ ‐Antitrypsin Deficiency: An Under‐Recognized Genetic Disorder,” Allergy 73, no. 11 (2018): 2110–2121, 10.1111/all.13558. 29984428 PMC6282978

[199] C. H. Tsai , A. C. Y. Lai , Y. C. Lin , et al., “Neutrophil Extracellular Trap Production and CCL4L2 Expression Influence Corticosteroid Response in Asthma,” Science Translational Medicine 15, no. 699 (2023): eadf3843, 10.1126/scitranslmed.adf3843. 37285400

[200] S. Shak , “Aerosolized Recombinant Human DNase I for the Treatment of Cystic Fibrosis,” Chest 107, no. 2 (1995): 65S–70S, 10.1378/chest.107.2_supplement.65s. 7842816

[201] N. Mayer‐Hamblett , F. Ratjen , R. Russell , et al., Randomized, Open‐Label Non‐Inferiority Trials Evaluating Discontinuation Versus Continuation of Hypertonic Saline or Dornase Alfa in Modulator Treated People With Cystic Fibrosis: Results From The Simplify Study, (2024). 10.1016/S2213-2600(22)00434-9 PMC10065895 36343646

[202] B. J. Tarrant , C. Le Maitre , L. Romero , et al., “Mucoactive Agents for Chronic, Non‐Cystic Fibrosis Lung Disease: A Systematic Review and Meta‐Analysis,” Respirology 22, no. 6 (2017): 1084–1092, 10.1111/resp.13047. 28397992

[203] J. Chen , T. Wang , X. Li , et al., “DNA of Neutrophil Extracellular Traps Promote NF‐κB‐Dependent Autoimmunity via cGAS/TLR9 in Chronic Obstructive Pulmonary Disease,” Signal Transduction and Targeted Therapy 9, no. 1 (2024): 163, 10.1038/s41392-024-01881-6. 38880789 PMC11180664

[204] G. K. Gupta and D. K. Agrawal , “CpG Oligodeoxynucleotides as TLR9 Agonists: Therapeutic Application in Allergy and Asthma,” BioDrugs 24, no. 4 (2010): 225–235, 10.2165/11536140-000000000-00000. 20623989

[205] A. Chakraborty , S. Tannenbaum , C. Rordorf , et al., “Pharmacokinetic and Pharmacodynamic Properties of Canakinumab, a Human Anti‐Interleukin‐1b Monoclonal Antibody,” Clinical Pharmacokinetics (2012). 10.2165/11599820-000000000-00000 PMC3584253 22550964

[206] P. M. O’Byrne , H. Metev , M. Puu , et al., “Efficacy and Safety of a CXCR2 Antagonist, AZD5069, in Patients With Uncontrolled Persistent Asthma: A Randomised, Double‐Blind, Placebo‐Controlled Trial,” Lancet Respiratory Medicine 4, no. 10 (2016): 797–806, 10.1016/s2213-2600(16)30227-2. 27574788

[207] A. De Soyza , I. Pavord , J. S. Elborn , et al., “A Randomised, Placebo‐Controlled Study of the CXCR2 Antagonist AZD5069 in Bronchiectasis,” European Respiratory Journal 46, no. 4 (2015): 1021–1032, 10.1183/13993003.00148-2015. 26341987

[208] B. F. Marple , “Allergic Rhinitis and Inflammatory Airway Disease: Interactions Within the Unified Airspace,” American Journal of Rhinology & Allergy 24, no. 4 (2010): 249–254, 10.2500/ajra.2010.24.3499. 20819460

[209] G. Ciprandi , L. M. Bellussi , G. C. Passali , V. Damiani , and D. Passali , “HMGB1 in Nasal Inflammatory Diseases: A Reappraisal 30 Years After Its Discovery,” Expert Review of Clinical Immunology 16, no. 5 (2020): 457–463, 10.1080/1744666x.2020.1752668. 32252560

[210] W. Huang , X. Chen , Z. Liu , et al., “Sphk1 Regulates HMGB1 via HDAC4 and Mediates Epithelial Pyroptosis in Allergic Rhinitis,” World Allergy Organization Journal 17, no. 9 (2024): 100963, 10.1016/j.waojou.2024.100963. 39295955 PMC11408713

[211] J. Ning and L. Qiao , “The Role of Necroptosis in Common Respiratory Diseases in Children,” Frontiers in Pediatrics 10 (2022): 945175, 10.3389/fped.2022.945175. 35967568 PMC9367635

[212] A. Jo and D. W. Kim , “Neutrophil Extracellular Traps in Airway Diseases: Pathological Roles and Therapeutic Implications,” International Journal of Mathematics and Statistics 24, no. 5 (2023): 5034, 10.3390/ijms24055034. PMC10003347 36902466

